# Longitudinal analysis of retinal cell state transitions in *RB1*-deficient retinal organoids reveals the nascent cone precursors are the earliest cell-origin of human retinoblastoma

**DOI:** 10.1038/s41419-025-08191-x

**Published:** 2026-01-14

**Authors:** Ke Ye, Yuan Wang, Ping Xu, Bingbing Xie, Shijing Wu, Wenxin Zhang, Guanjie Gao, Dandan Zheng, Xiaojing Song, Suai Zhang, Fuying Guo, Yongping Li, Yizhi Liu, Jie Wang, Ruifang Sui, Xiufeng Zhong

**Affiliations:** 1 State Key Laboratory of Ophthalmology, Zhongshan Ophthalmic Center, Sun Yat-sen University, Guangdong Provincial Key Laboratory of Ophthalmology and Visual Science, Guangzhou, China; 2 Department of Ophthalmology, Peking Union Medical College Hospital, Chinese Academy of Medical Sciences, Beijing, China; 3 CAS Key Laboratory of Regenerative Biology, Guangdong Provincial Key Laboratory of Stem Cell and Regenerative Medicine, Guangzhou Institutes of Biomedicine and Health, Chinese Academy of Sciences, Guangzhou, China; 4 State Key Laboratory of Respiratory Disease, Guangzhou Institutes of Biomedicine and Health, Chinese Academy of Sciences, Guangzhou, China; 5 China-New Zealand Joint Laboratory on Biomedicine and Health, Guangzhou, China

**Keywords:** Induced pluripotent stem cells, Eye cancer

## Abstract

All cancers arise from the malignant transformation of normal cells, yet their cells-of-origin remain challenging to identify due to the inability to directly observe dynamic changes in human tumors. Retinoblastoma (Rb), a malignant intraocular cancer, serves as a well-established model for investigating the molecular and cellular mechanisms underlying tumorigenesis. While the maturing cone precursors (CPs) have been proposed as the cellular origin of human Rb, it is unclear whether other retinal cell types are similarly sensitive to *RB1* inactivation. In this study, we developed *RB1*-deficient human retinal organoids (ROs) models using *RB1*^−/−^ or *RB1*^+/-^ human induced pluripotent stem cells (hiPSCs). *RB1*^−/−^ hiPSCs generated tumor cells that recapitulated key features of human Rb and formed serial orthotopic xenografts. Importantly, *RB1* loss induced overproliferation of ATOH7^+^ neurogenic retinal progenitor cells (nRPCs), which disrupted retinal development by generating ectopic dividing early-born retinal cells (retinal ganglion cells and CPs). Single-cell RNA sequencing analysis confirmed that ATOH7^+^/RXRγ^+^ nascent CPs survived and ultimately drove Rb tumorigenesis. In contrast, monoallelic *RB1* inactivation resulting in low pRB expression did not induce proliferation of nascent CPs, but only triggered overproliferation of nRPCs, leading to a retinocytoma-like phenotype. Finally, a potential therapeutic target for Rb was identified from multi-omics data and validated through knockdown experiment and a small-molecule inhibitor. Our findings demonstrate, for the first time, that nRPCs are the most sensitive cells to *RB1* loss inducing abnormal proliferation of nascent retinal cells, while ATOH7^+^ nascent CPs represent the earliest cellular origin of human Rb. These insights may facilitate the development of targeted therapies for Rb.

## Introduction

Retinoblastoma (Rb) is a pediatric ocular malignancy originating in the developing retina [1]. The majority of Rb cases result from biallelic inactivation of *RB1*, the first identified tumor suppressor gene [2]. This gene encodes the RB protein (pRB), which regulates the G1/S phase transition of the cell cycle by modulating the activity of E2F transcription factors [3]. Through its roles in controlling cell-cycle progression and cell differentiation, pRB plays a critical role in suppressing tumorigenesis [4, 5]. However, pRB exhibits cell-type-specific and stage-dependent expression patterns during retinogenesis [6], and this complexity has hindered a complete understanding of pRB biology in the human retina, including the timing and mechanisms initiating tumorigenesis.

Identifying the cellular origin of Rb has long remained a pivotal and challenging question. Previous analyses with human Rb specimens suggest that Rb may arise from retinal progenitor cells (RPCs), photoreceptor cells (PRCs) or interneurons, given the high molecular and cellular heterogeneity of Rb tumors [7–11]. However, most clinical samples are obtained during late-stage of tumor progression, where the observed cell types may not accurately reflect the original cells that acquire cancer-initiating mutations and drive overt tumor growth. This limitation complicates efforts to determine when or how tumorigenesis begins. Studies using short hairpin RNA (shRNA)-mediated *RB1* inactivation in human fetal retinas have proposed that ARR3^+^ maturing cone precursors (CPs) are potential cells-of-origin [9, 10]. Nevertheless, whether other retinal cell types can drive *RB1*-deficient tumor development remains unclear. Additionally, mouse models with *RB1* loss fail to recapitulate key features of human Rb due to species-specific differences [8, 12, 13], underscoring the need for a novel, accessible and reproducible model of human retinogenesis: retinal organoids (ROs) [14].

ROs, derived from pluripotent stem cells, recapitulate human retinogenesis in vitro [15]. During RO differentiation, all seven major retinal cell subtypes-retinal ganglion cells (RGCs), cones, horizontal cells (HCs), amacrine cells (ACs), Müller glia (MGs), bipolar cells (BCs) and rods-originate from RPCs in a temporally ordered manner [15, 16]. Several studies have attempted to model human Rb using ROs, yielding inconsistent results. Zheng et al. observed cell apoptosis without tumors in *RB1*-deleted ROs [17]. Norrie et al. developed orthotopic Rb xenografts by injecting *RB1*-mutated cells derived from day 45 ROs, and proposing that Rb tumors arise from progenitor-signature cells [18]. Jin’s group observed Rb formation in *RB1*-null ROs by day 120, which generated orthotopic xenografts in mice, and identified maturing CPs as the cellular origin of Rb [19]. Rozanska et al. reported a comparable cell-of-origin [20]. However, recent evidence implicates that early-stage CPs may contribute to Rb initiation [21], highlighting the need for detailed analysis to clarify the specific cellular states and their contributions to Rb development.

In this study, we established pRB-negative and pRB-reduced RO models using *RB1*^−/−^ or *RB1*^+/-^ hiPSCs and characterized the malignant transformation of normal retinal cells throughout retinogenesis. Only ROs with complete loss of *RB1* developed Rb tumors that mimicked key features of human Rb and formed serial orthotopic xenografts in mice. Our findings revealed that ATOH7^+^ neurogenic RPCs (nRPCs) were the most sensitive cell types to *RB1* loss, driving abnormal proliferation of nascent postmitotic retinal cells, and the ATOH7^+^/RXRγ^+^ nascent CPs represented the earliest cellular origin of human Rb. In contrast, the monoallelic *RB1* inactivation did not disturb cell division in nascent CPs, but induced nRPCs overproliferation, leading to a retinocytoma-like lesion. In addition, a multi-omics analysis identified a potential therapeutic target for Rb, which we validated using shRNA and small-molecule inhibitors. These insights advance our understanding of the cellular and molecular mechanisms underlying human Rb and may facilitate therapeutic development.

## Results

### *RB1* deletion induces tumor formation of Rb in ROs

Two clones of *RB1*^−/−^ hiPSC lines (C1 and C5) were generated using CRISPR/Cas9 technology (Fig. S1A, B), exhibiting reduced pRB protein levels but retaining pluripotency (Fig. S1C-H). We did not identify differences in cell cycle and the potential to form teratomas between WT and *RB1*^−/−^ hiPSCs (Fig. S2A-C). To generate *RB1*-inactivated hiPSCs under different genetic backgrounds, another knockout strategy was performed to generate *RB1*-inactivated hiPSCs from another hiPSC line (Fig. S3A). One *RB1* biallelic-deleted hiPSC line (*RB1*^Puro/Puro^) and one *RB1* heterozygous-deleted hiPSC line (*RB1*^W/Puro^) were obtained (Fig. S3B-F).

**Fig. 1:**
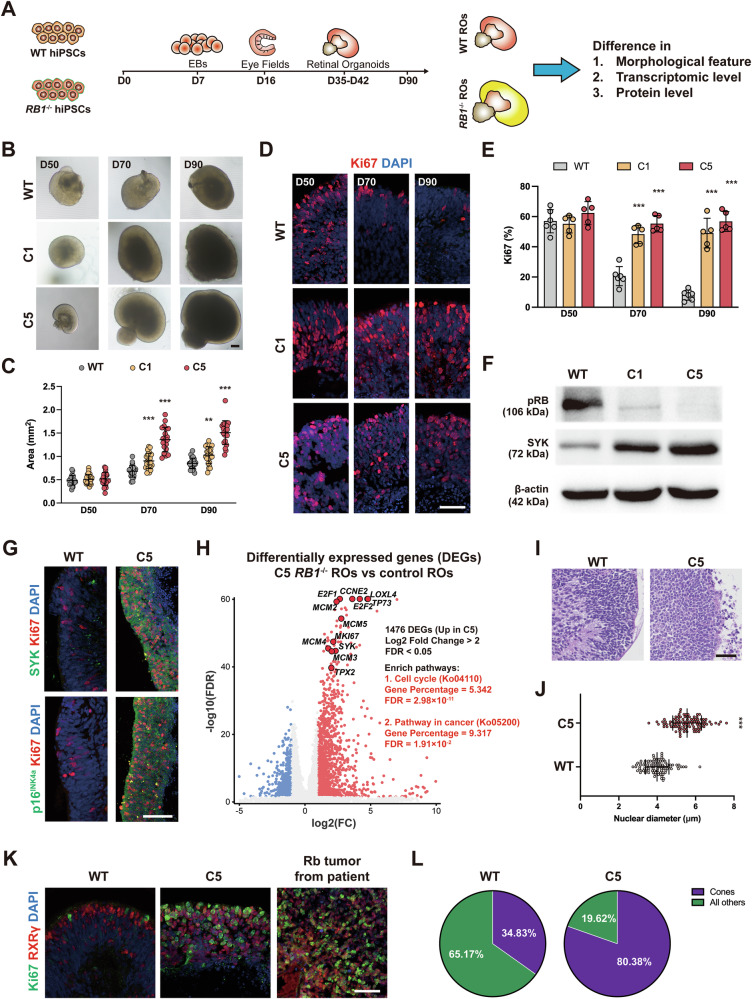
Tumorigenesis of ROs with *RB1* loss in vitro*.* **A** Experimental design for characterizing *RB1*^−/−^ ROs. **B** Representative microscopic images of WT and *RB1*^−/−^ ROs from day 50 to day 90. **C** Quantification of size of ROs in different culture phase. Data represent mean ± SD. *** *P* < 0.001 vs. WT, *n* = 23 (WT), *n* = 24 (C1), *n* = 22 (C5). **D** Representative immunostaining for Ki67 in WT and *RB1*^−/−^ ROs from day 50 to 90. **E** Quantification of Ki67^+^ cells from day 50 to 90. Data represent mean ± SD. *** *P* < 0.001 vs. WT, *n* = 6 (WT), *n* = 5 (C1), *n* = 5 (C5). **F** Western blot analysis of pRB and SYK protein level in WT and *RB1*^−/−^ ROs at day 90. **G** Representative immunostaining for SYK, p16^INK4a^ and Ki67 in WT and *RB1*^−/−^ ROs at day 90. **H** Volcano plot visualization of DEGs and related enrich pathways from bulk RNA-seq data obtained between WT and C5 *RB1*^−/−^ ROs. **I** Representative H&E staining of WT and C5 *RB1*^−/−^ ROs at day 80. **J** Quantification of nuclear size of WT and C5 *RB1*^−/−^ ROs. Data represent mean ± SD. *** *P* < 0.001 vs. WT, *n* = 100. **K** Representative immunostaining for Ki67 and RXRγ in WT, C5 *RB1*^−/−^ ROs and patient tumor sample. **L** Quantification of RXRγ^+^ cells in WT and C5 *RB1*^−/−^ ROs. Scale bars = 200 μm (**B**) and 50 μm (**D**, **G**, **I**, **K**).

RO differentiation was performed with our optimized protocols (Fig. 1A) [15, 22]. No significant disorders were observed from embryoid body to early ROs (Fig. S4A), while pRB^+^ cells were detected only in WT ROs (Fig. S4B). By 70 days, *RB1*^−/−^ ROs grew larger than WT ROs (Fig. 1B, C), with increased Ki67^+^ and EdU^+^ cells (Figs. 1D, E, S4C, D). Rb markers SYK and p16^INK4a^ were detected only in *RB1*^−/−^ ROs at day 90 (Figs. 1F, G, S4E). Transcriptomic analysis revealed the upregulation of cell cycle- (*MKI67*, *CCNE2* etc.) and cancer-related genes (*SYK*, *MCM2* etc.) (Fig. 1H), and GSEA confirmed the enrichment of Rb-related pathways (Fig. S4G). Morphologically, *RB1*^−/−^ ROs lost laminated structure, comprised homogenous tumor-like cells with a larger nuclear size and mitotic figures (Fig. 1I, J, S4F). Most cells in *RB1*^−/−^ ROs expressed the CP marker RXRγ, resembling clinical Rb (Figs. 1K, L, S4H, I). Similar phenomenon was identified in other *RB1* homozygous mutated hiPSCs-derived ROs (Fig. S5). Taken together, these results demonstrate that *RB1* loss can robustly induce tumorigenesis of cone-rich Rb in RO model.

### Orthotopic Rb xenografts are successively generated from *RB1*^−/−^ ROs in immunodeficient mice

**Fig. 2:**
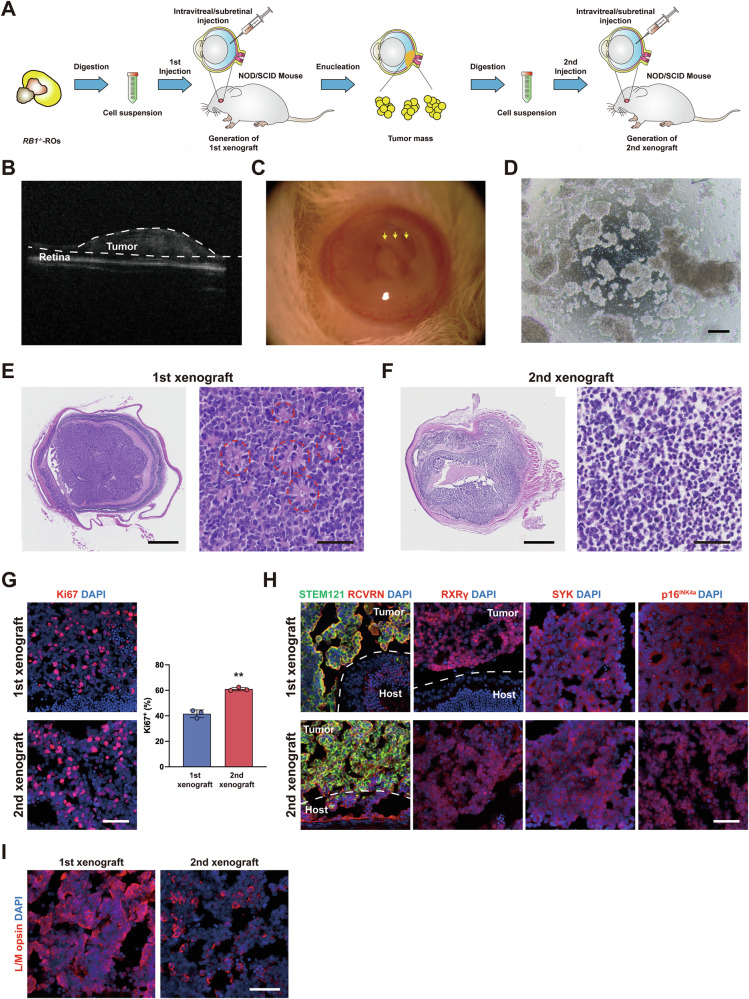
Xenograft of ROs-derived cells into mice in vivo*.* **A** Schematic outline of the generation of 1st and 2nd xenograft. **B** Representative optical coherence tomography (OCT) images of engrafted eyes at 10 weeks from *RB1*^−/−^ ROs. An obvious highly reflective tumor was observed. **C** Representative slit-lamp images of engrafted eyes at 13 weeks from *RB1*^−/−^ ROs. Whitish tumor cells were observed in anterior chambers. **D** Representative microscopic image of tumor mass derived from 1st xenograft. **E** Representative H&E staining images of 1st xenograft from *RB1*^−/−^ ROs. The dash lines indicate the rosette structure. **F** Representative H&E staining images of 2nd xenograft with injection of 1st tumor cells. No rosettes were observed. **G** Representative immunostaining for Ki67 and quantification of Ki67^+^ cells in 1st and 2nd xenograft. ** *P* < 0.01 vs. 1st xenograft, *n* = 3. **H** Representative immunostaining for human cells specific marker (STEM121), cone markers (RCVRN, RXRγ) and Rb markers (SYK, p16^INK4a^) in 1st and 2nd tumor. **I** Representative immunostaining for L/M opsin in 1st and 2nd tumors. Scale bars = 200 μm (**D**), 500 (left), 50 (right) μm (**E**, **F**) and 50 μm (**G**, **H**, **I**).

Since xenograft tumor formation is the golden standard to confirm malignant feature of cells, we investigated whether retinal tumor-like cells from *RB1*^−/−^ ROs recapitulated the formation and characteristics of human Rb in mice eyes [23]. Cells from D70 *RB1*^−/−^ ROs were engrafted into the intravitreal or subretinal spaces of NOD/SCID mice to generate primary xenograft tumors (Fig. 2A). 10 weeks after injection (WAI), SD-OCT identified highly reflective masses in some mice (Fig. 2B). 13 WAI, anterior chambers of mice eyes were full of whitish tumor cells (Fig. 2C). After enucleation, tumor cells were identified in 7 out of 10 mice in *RB1*^−/−^ group (Fig. 2D, Table S1). The tumor cells formed numerous Flexner-Wintersteiner (F-W) rosettes with mitosis figures, resembling well-differentiated human Rb (Figs. 2E, S6A). Transmission electron microscopy (TEM) analysis confirmed the F-W rosettes, high nucleocytoplasmic ratio and abundant mitochondria in tumor cells (Fig. S6B). In contrast, no tumors formed in WT group (Fig. S6C-E).

To further explore the tumorigenicity, cells from primary xenograft were injected into the eyes of five additional mice. All mice developed eye tumors 13 WAI (Table S2). Surprisingly, the 2nd xenograft tumors lacked rosette structures, but presented necrosis region (Fig. 2F). Immunostaining analysis confirmed the higher ratio of Ki67^+^ cells in the 2nd xenograft (Fig. 2G). These features were more similar to poorly-differentiated human Rb or xenograft tumors from Rb cell lines (Fig. S6A, F). Both of the 1st and 2nd xenograft tumors expressed cone and Rb markers (Fig. 2H), but not the markers specific for other retinal cell types (Fig. S6G). L/M opsin was highly expressed in 1st xenograft but low in the 2nd xenograft, consistent with their high- and low-differentiated states, respectively (Fig. 2I). These results demonstrate that retinal tumor-like cells from *RB1*^−/−^ ROs have the ability to develop into the typical phenotype of Rb tumors with strong expansion capacity.

### *RB1* loss induces overproliferation of nRPCs, generating proliferative nascent retinal cells in *RB1*^−/−^ ROs

**Fig. 3:**
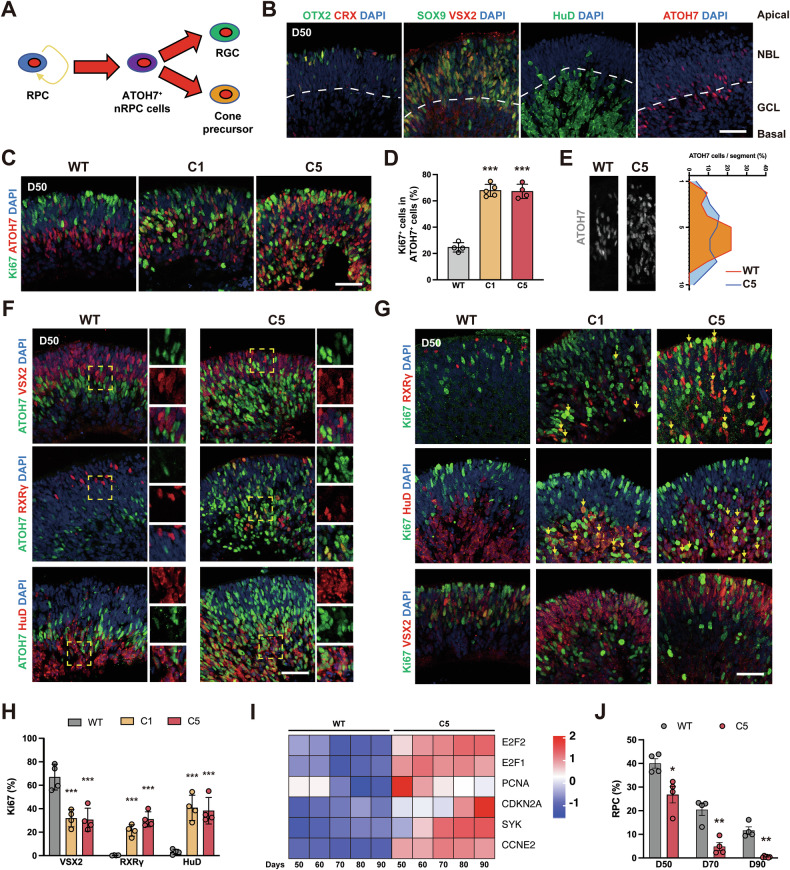
pRB-mediated proliferation defects in nRPCs disrupt late-stage retinogenesis. **A** Summary of the differentiation of early-born retinal cells from ATOH7^+^ nRPC cells. **B** Representative immunostaining for early PRC markers (OTX2, CRX), RPC markers (VSX2, SOX9), RGC marker (HuD) and ATOH7 in WT ROs at day 50 showing the localization of each type of cells. *NBL* neuroblastic cell layer; *GCL* ganglion cell layer. **C** Representative immunostaining for Ki67 and ATOH7 in WT and *RB1*^−/−^ ROs at day 50. **D** Quantification of Ki67^+^ cells within ATOH7^+^ cells in WT and *RB1*^−/−^ ROs at day 50. Data represent mean ± SD. *** *P* < 0.001 vs. WT, *n* = 4 (WT), *n* = 5 (C1), *n* = 4 (C5). **E** Representative immunostaining for ATOH7 and relative distribution of ATOH7^+^ cells in WT and *RB1*^−/−^ ROs at day 50. **F** Representative immunostaining for VSX2, RXRγ, HuD and ATOH7 in WT and *RB1*^−/−^ ROs at day 50. **G** Representative immunostaining for VSX2, RXRγ, HuD and Ki67 in WT and *RB1*^−/−^ ROs at day 50. Arrows indicate the Ki67^+^ cells in different cell types. **H** Quantification of Ki67^+^ cells within RXRγ^+^, HuD^+^ and VSX2^+^ cells in WT and *RB1*^−/−^ ROs at day 50. Data represent mean ± SD. *** *P* < 0.001 vs. WT, *n* = 4. **I** Heatmap of differential expression of Rb marker genes in WT and *RB1*^−/−^ ROs from day 50 to 90. **J** Cell ratio of RPC (VSX2^+^, Ki67^+^ cells) from day 50 to day 90. Data represent mean ± SD. * *P* < 0.05, ** *P* < 0.01 vs. WT, *n* = 4. Scale bars = 50 μm (**B**, **C**, **F**, **G**).

Here, we explore how *RB1* deletion affects retinal development in ROs. During early retinogenesis, RPCs gradually develop into ATOH7^+^ nRPCs, which generate CPs and RGCs (Fig. 3A) [24]. In WT ROs, ATOH7^+^ cells were mostly located in the neuroblast layer (NBL) and ganglion cell layer (GCL) (Fig. 3B). In *RB1*^−/−^ ROs, ATOH7^+^ cells showed disordered layer distribution with elevated proliferation, as evidenced by Ki67^+^ and EdU^+^ cells count (Figs. 3C–E, S7A-D). It induced more ATOH7^+^ cells in *RB1*^−/−^ ROs from day 35 (Fig. S7E). In nRPCs-derived offsprings, a higher proportion of ATOH7^+^ cells co-expressed with RXRγ or HuD in *RB1*^−/−^ ROs (Fig. 3F), implying that more CPs and RGCs were generated. Notably, the *RB1*^−/−^ CPs and RGCs failed to exit cell cycle (Fig. 3G, H). RNA-seq and immunostaining analysis revealed that *RB1*-targetted genes were significantly upregulated in *RB1*^−/−^ ROs starting at day 50 (Figs. 3I, S7F), indicating that pRB loss perturbs cell cycle regulation during early retinogenesis.

In contrast, the percentage of Ki67^+^/VSX2^+^ RPCs declined rapidly at day 50 (Fig. 3G, H). EdU labeling corroborated this finding (Fig. S7G, H). This led to a marked reduction of RPC in *RB1*^−/−^ ROs, as indicated by both cell ratio and mRNA level (Figs. 3J, S7I, J). We speculate that the reduction of RPC population may reflect the increase in other dividing cell populations, such as ectopic dividing CPs and RGCs, and/or the death of RPCs in *RB1*^−/−^ ROs. Further studies will be needed to explore the potential mechanism. Thus, *RB1* loss enhances the proliferation of nRPC-derived nascent CPs and RGCs, but also potentially disrupts RPC survival.

### Longitudinal analysis shows that pRB loss induced CPs tumor-like clonal growth at the expense of specification of late born retinal cells

**Fig. 4:**
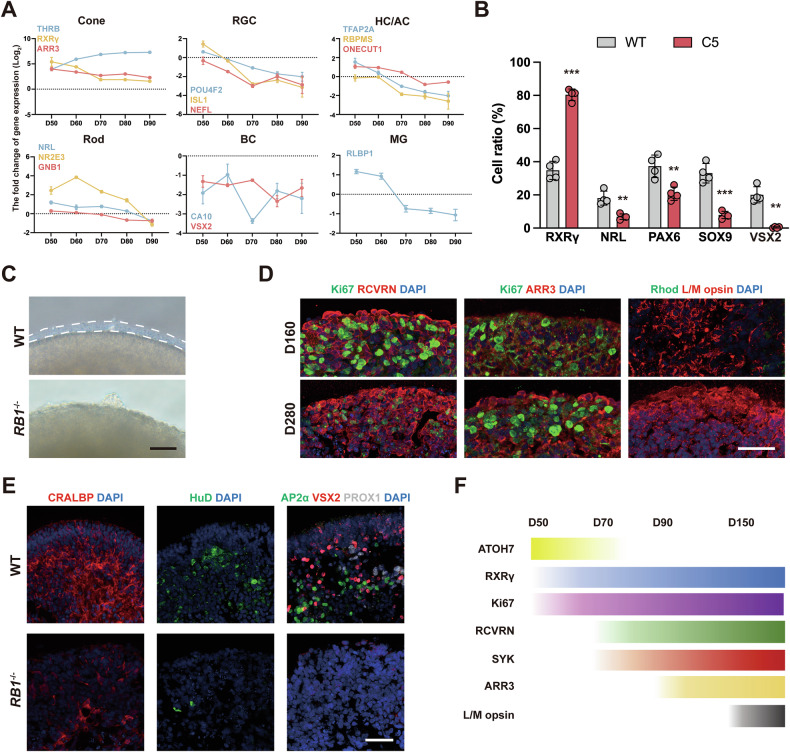
Longitudinal analysis of pRB-null ROs. **A** The fold change of expression of cell type-specific marker genes from RNA-seq in WT and *RB1*^−/−^ ROs from day 50 to 90, *n* = 3. **B** Quantification of the ratio of RXRγ^+^, NRL^+^, SOX9^+^, PAX6^+^ and VSX2^+^ cells in WT and *RB1*^−/−^ ROs at day 90. Data represent mean ± SD. ** *P* < 0.01, *** *P* < 0.001 vs. WT, *n* = 4. **C** Representative microscopic images of WT and *RB1*^−/−^ ROs at day 160. **D** Representative immunostaining for RCVRN, ARR3, Ki67, L/M opsin and Rhodopsin in *RB1*^−/−^ ROs at day 160 and 280. **E** Representative immunostaining for CRALBP^+^ (MGs), HuD^+^ (RGCs), AP2α^+^ (HCs), PROX1^+^ (ACs), and VSX2^+^ (BCs) in WT and RB1^−/−^ ROs at day 280. **F** Summary of temporal expression of nRPC, cone and Rb markers during tumor development. Scale bars = 50 μm (**D**, **E**) and 200 μm (**C**).

During retinal development of *RB1*^−/−^ ROs, the expression of retinal cell-specific genes other than those specific to cones was reduced, including RGC-specific genes (Fig. 4A). *RB1* loss also induced apoptosis in cells by day 70, as evidenced by immunostaining for cleaved caspase-3 (CC3)^+^ (Fig. S8A, B). pRB-negative RGCs showed greater susceptibility to apoptosis than CPs (Fig. S8C, D). Immunostaining analysis for multiple retinal markers at day 90 also confirmed that pRB-negative ROs failed to develop into other retinal cell types other than cones, which displayed tumor-like clonal expansion (Figs. 4B, S9A, B).

To track long-term tumor progression, *RB1*^−/−^ ROs were cultured up to day 280. Unlike WT ROs that RCVRN^+^ photoreceptor precursors developed into L/M opsin^+^ and Rhodopsin^+^ mature photoreceptors (Fig. S9C) [15, 25, 26], *RB1*^−/−^ ROs lacked outer segments formation and expressed L/M opsin, SYK and Ki67 in the same period without Rhodopsin^+^ rods (Figs. 4C, D, S9D). Additionally, populations of other retinal cells were hardly detectable in *RB1*^−/−^ ROs (Fig. 4E). This cellular architecture was consistent with that of Rb tumor sample. In conclusion, the above results demonstrate that pRB loss drives the proliferation of death-resistant CPs, impairing late retinal differentiation while promoting tumorigenesis (Fig. 4F).

### Single-cell transcriptomic profiling reveals nascent CPs are the earliest source of Rb tumor in *RB1*^−/−^ RO models

**Fig. 5:**
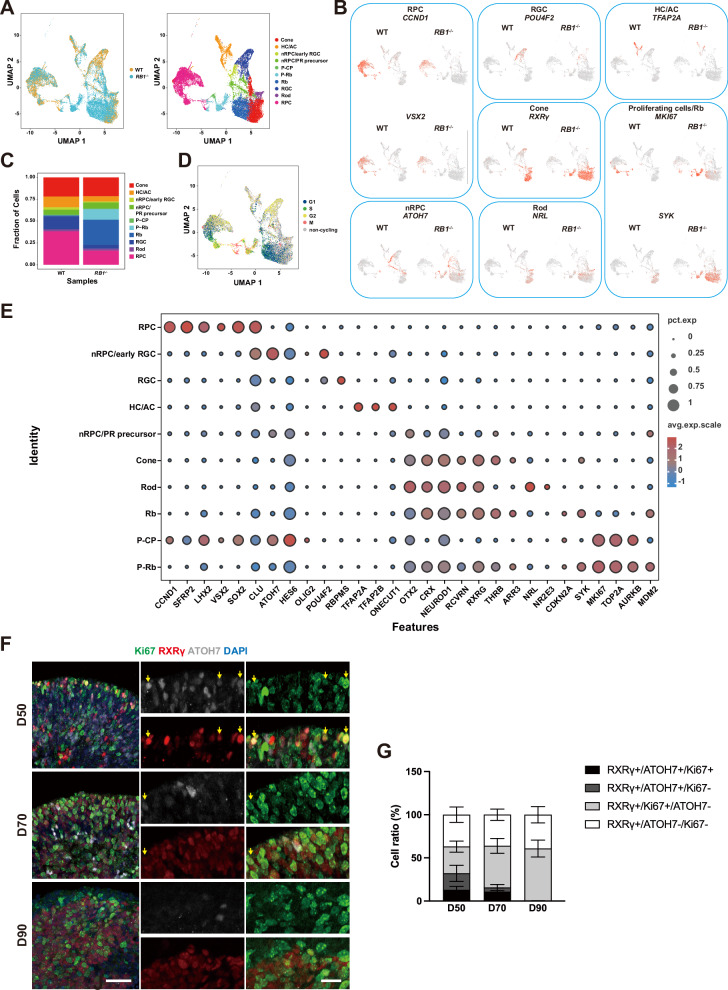
Single-cell RNA sequencing identifies nascent CPs as the cellular origin of Rb. **A** UMAP plot of unsupervised cell cluster of WT and *RB1*^−/−^ ROs at day 80. Different colors correspond to different cell types. **B** Feature plots displaying expression profiles of retinal cell-type markers and Rb-specific markers. **C** Quantification of cell ratio in different cell clusters. **D** Distribution of cells in different cell cycle. **E** Dot plot visualization of expression of different cell type-specific markers. **F** Representative immunostaining for Ki67, RXRγ and ATOH7 in *RB1*^−/−^ ROs from day 50 to day 90. Arrows indicate triple positive cells at different culture day. **G** Quantification of ATOH7^+^ and Ki67^+^ cells in RXRγ^+^ cells in *RB1*^−/−^ ROs from day 50 to day 90, *n* = 4. Scale bars = 50 μm (**F**).

ScRNA-seq was performed to analyze the cellular composition and developmental trajectory of Rb in *RB1*^−/−^ ROs. After excluding cell cycle genes to prevent bias, cells were classified by different markers: RPCs (*VSX2*/*CCND1*), nascent RGCs (*ATOH7*/*POU4F2*), RGCs (low *ATOH7*/high *POU4F2*), nascent pan-PRC precursor (*ATOH7*/*OTX2*), HCs/ACs (*TFAP2A*), Rods (*NRL*), and nascent cones (*ATOH7*/*RXRG*) (Figs. 5A, B, E, S10A). *RB1*^−/−^ ROs showed decreasing cell number of RPCs (Fig. 5B, C) with three additional cell populations, including a proliferative subtype of ATOH7^+^ cells, which highly expressed cone-determination genes including *Olig2* and *Onecut1* [27]. We defined these cells as proliferating nascent CP (P-CP) (Figs. 5A–C, E, S10B, C). This cell population was also identified by immunostaining using triple labeling starting at day 50 (Fig. 5F, G). Since ATOH7 is expressed only in the committed stage of CPs [24, 28], these results suggest that the nascent CPs, which are transient population, are sensitive to *RB1* inactivation. A second population expressed cone and Rb-related genes (*SYK*, *CDKN2A*, *MDM2*) with lower level of *MKI67*, and was considered as non-proliferative Rb cells (Rb). The proliferative Rb cells (P-Rb) showed higher levels of *MKI67* and Rb-related genes (*SYK*, *TOP2A*, *MDM2*) (Figs. 5B, C, E, S10B). Both population re-entered cell cycle (Figs. 5D, S10C), and enriched cell cycle- and tumor-related pathways versus other retinal cells (Fig. S10D).

Next, we explored how the P-CPs in *RB1*^−/−^ ROs transitioned into Rb using pseudotime trajectory analysis with Monocle 2. The data showed that most RPCs presented at the beginning of trajectory, while P-CPs positioned terminally adjacent to P-Rb (Fig. S11A, B). These results suggest that these cells may develop directly into proliferative Rb cells. The pseudo-time heatmap showed the progressive dynamic expression of selected Rb genes and cell cycle-related genes (Fig. S11C). Taken together, these data confirm that malignant Rb-related cells arise from the committed or nascent CPs.

### Low pRB expression results in a retinocytoma-like phenotype in *RB1*^+/-^ ROs

Two hiPSC with heterozygous *RB1* mutation were used to confirm the necessity of biallelic *RB1* loss in Rb initiation. One hiPSC line (*RB1*^W/Puro^) exhibited pRB protein level similar to WT hiPSCs (Fig. S3B). The second line was generated by reprogramming somatic cells from a patient carrying a heterozygous *RB1* mutation (c.658C>G, p.Leu220Val) (Fig. S12A-C) [29]. Sanger sequencing confirmed the presence of the same mutation in hiPSCs (Fig. S12A). mRNA splicing analysis was performed on patient-specific hiPSCs to assess the pathogenic potential of this mutation (Fig. S12D). PCR products revealed two alternatively spliced mRNA variants: partial exon 7 or full exon 8 skipping (Fig. S12E, F), leading to a reduction of pRB protein level (Fig. S12G, H).

**Fig. 6:**
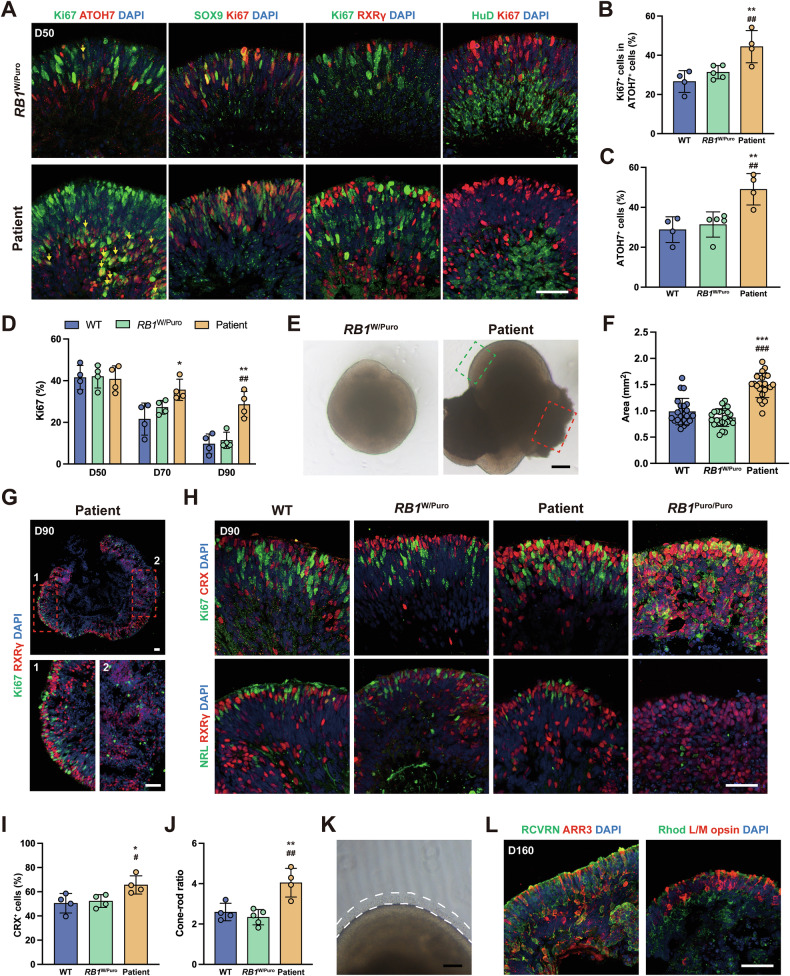
pRB protein level-dependent developmental defects in *RB1*^+/−^ ROs. **A** Representative immunostaining for SOX9, RXRγ, HuD and Ki67 in *RB1*^W/Puro^ and patient-specific ROs at day 50. Arrows indicate the Ki67^+^ cells in ATOH7^+^ cells. **B** Quantification of Ki67^+^ cells within ATOH7^+^ cells in different types of ROs at day 50. Data represent mean ± SD. ** *P* < 0.01 vs. WT; ## *P* < 0.01 vs. *RB1*^W/Puro^, *n* = 4 (WT), *n* = 5 (*RB1*^W/Puro^), *n* = 4 (Patient). **C** Quantification of the ratio of ATOH7^+^ cells in different types of ROs at day 50. ** *P* < 0.01 vs. WT; ## *P* < 0.01 vs. *RB1*^W/Puro^, *n* = 4 (WT), *n* = 5 (*RB1*^W/Puro^), *n* = 4 (Patient). **D** Quantification of Ki67^+^ cells from day 50 to 90. Data represent mean ± SD. * *P* < 0.05 vs. WT; ** *P* < 0.01 vs. WT; ## *P* < 0.01 vs. *RB1*^W/Puro^, *n* = 4. **E** Representative microscopic images of WT, *RB1*^W/Puro^ and patient-specific ROs at day 90. Dashed green box indicates the neural retina. Dashed red box indicates the retinal lesion in patient-specific ROs. **F** Quantification of size of different types of ROs at day 90. Data represent mean ± SD. *** *P* < 0.001 vs. WT; ### *P* < 0.001 vs. *RB1*^W/Puro^, *n* = 22 (WT), *n* = 24 (*RB1*^W/Puro^), *n* = 22 (Patient). **G** Representative immunostaining for RXRγ and Ki67 in patient-specific ROs at day 90. Region 1 indicates the normal neural retina. Region 2 indicates the disorder cell mass. **H** Representative immunostaining for CRX, Ki67, RXRγ and NRL in WT, *RB1*^W/Puro^, patient-specific and *RB1*^Puro/Puro^ ROs at day 90. **I** Quantification of the ratio of CRX^+^ cells in different types of ROs at day 90. Data represent mean ± SD.* *P* < 0.05 vs. WT; # *P* < 0.05 vs. *RB1*^W/Puro^, *n* = 4. **J** Quantification of the ratio of Cones/Rods in different types of ROs at day 90. Data represent mean ± SD. ** P < 0.01 vs. WT; ## *P* < 0.01 vs. *RB1*^W/Puro^, *n* = 4 (WT), *n* = 5 (*RB1*^W/Puro^), *n* = 5 (Patient). **K** Representative microscopic images of patient-specific ROs at day 160. **L** Representative immunostaining for RCVRN, ARR3, Rhodopsin and L/M opsin in patient-specific ROs at day 160. Scale bars = 50 μm (**A**, **G**, **H**, **L**), 200 μm (**E**) and 100 μm (**K**).

When induced into ROs, a higher ratio of Ki67^+^ cells within ATOH7^+^ cells was identified in patient-specific ROs at day 50 (Fig. 6A, B), with additional ATOH7^+^ cells also identified (Fig. 6C). However, the low pRB level did not affect CPs, RGCs and RPCs proliferation (Fig. 6A, S13A). Since this mutation induced cell proliferation from day 70 to day 90, patient-specific ROs grew significantly larger than WT and *RB1*^W/Puro^ ROs at day 90 (Fig. 6D-F). In addition, retinal lesions were observed at high frequency in patient-specific ROs lacking proliferative CPs (Figs. 6G, S13B). These retinal lesions resembled the cellular features of retinocytoma [30]. In the normal region of retina, RPCs were present in both ROs at day 90 (Fig. S13C). Importantly, more photoreceptors and a higher cone-to-rod ratio were detected in patient-derived ROs (Fig. 6H–J). The normal region of retina eventually developed into mature stage, characterized by matured photoreceptors and other retinal cell types (Figs. 6K, L, S13D). These results demonstrate that while monoallelic loss of *RB1* is insufficient to induce CPs proliferation, the increased number of ATOH7^+^ nRPCs in *RB1*^+/-^ ROs lead to retinal lesions by generating more non-proliferative cones.

### Multi-omics analysis of *RB1*^−/−^ ROs identifies a potential therapeutic target

**Fig. 7:**
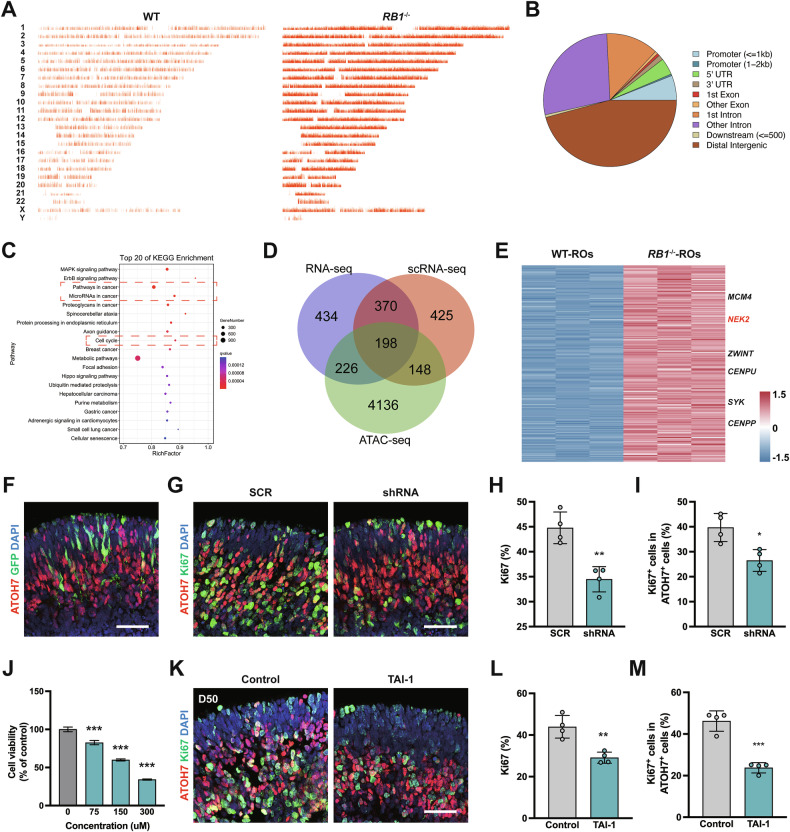
Exploration of potential therapeutic target for Rb from multi-omics analysis of *RB1*^−/−^ ROs. **A** Visualization of peaks over chromosomes in WT and *RB1*^−/−^ ROs. **B** Genome-wide distribution of accessible chromatin regions in *RB1*^−/−^ ROs. **C** Top 10 significantly enriched KEGG pathways among genes with accessible chromatin regions. **D** Venn diagram of consistently upregulated genes identified through integrated analysis of bulk RNA-seq (upregulated in *RB1*^−/−^ ROs), ATAC-seq (upregulated in *RB1*^−/−^ ROs), and scRNA-seq (upregulated in Rb cluster) data. **E** Heatmap of the collective up-regulated genes from three data sets in WT and *RB1*^−/−^ ROs. **F** Representative immunostaining for ATOH7 in *RB1*^−/−^ ROs after transfection of NEK2-shRNA lentivirus. The present of GFP^+^ cells indicated the successful of transduction. **G** Representative immunostaining for ATOH7 and Ki67 in *RB1*^−/−^ ROs after transfection of NEK2-shRNA lentivirus and SCR-shRNA lentivirus. **H** Quantification of Ki67^+^ cells in *RB1*^−/−^ ROs at day 50 after transfection of lentivirus. Data represent mean ± SD. ** *P* < 0.01 vs. control, *n* = 4. **I** Quantification of Ki67^+^ cells within ATOH7^+^ cells in *RB1*^−/−^ ROs after transfection of lentivirus. Data represent mean ± SD. ** *P* < 0.01 vs. SCR, *n* = 4. **J** Cell viability of Y79 cells after 72 h treatment with TAI-1 at different concentrations by CCK8. Data represent mean ± SD. *** *P* < 0.001 vs. control, n = 6. **K** Representative immunostaining for ATOH7 and Ki67 in *RB1*^−/−^ ROs at day 50 after treatment of TAI-1. **L** Quantification of Ki67^+^ cells in *RB1*^−/−^ ROs at day 50 after treatment of TAI-1. Data represent mean ± SD. ** *P* < 0.01 vs. control, n = 4. **M** Quantification of Ki67^+^ cells within ATOH7^+^ cells in *RB1*^−/−^ ROs at day 50 after treatment of TAI-1. Data represent mean ± SD. *** *P* < 0.001 vs. control, *n* = 4. Scale bars = 50 μm (**F**, **G**, **K**).

We explored potential therapeutic targets using multiple sequencing datasets generated from RO-derived Rb models. ATAC-seq was performed to assess the differences in chromatin accessibility [31]. *RB1*^−/−^ ROs exhibited more chromatin-accessible regions than WT ROs (Figs. 7A, S14A), while the majority of the open regions being localized to distal intergenic regions and introns (Fig. 7B). The differentially accessible region (DARs) associated genes were enriched in pathways related to cancer and cell cycle (Fig. 7C). In contrast, a smaller proportion of distal intergenic regions were accessible in WT ROs (Fig. S14B).

Next, three datasets were analyzed to explore potential therapeutic targets (Fig. S14C). After merging, 198 genes were commonly upregulated (Fig. 7D, E). Among these, 10 hub genes were identified, including *NEK2* (Figs. 7E, S14D, E). *NEK2* encodes a kinase involved in mitotic regulation [32]. Clinical Rb samples and human Rb cell line showed higher NEK2 expression level than normal retina (Fig. S14F, G). To validate the role of *NEK2* in Rb tumorigenesis, we performed knockdown experiment using lentivirus co-expressing *NEK2*-targeted shRNA and GFP [33]. Three shRNAs were designed and all significantly reduced the viability of Y79 (Fig. S15A, B) [34, 35]. Transfection of *RB1*^−/−^ ROs at day 55 with lentivirus expressing shRNA-2 for 24 h led to an inhibition of cell proliferation and decrease in the ratio of Ki67^+^ cells within ATOH7^+^ nRPCs 3 days later (Fig. 7F–H). Despite the ratio of ATOH7^+^ cells unchanged, *NEK2* knockdown decreased the ratio of Ki67^+^ cell and early-born CPs (RXRγ^+^/ATOH7^+^) within ATOH7^+^ nRPCs (Figs. 7I, S15C-E), suggesting that downregulation of *NEK2* contributed to suppress ectopic proliferation in early-born CPs in *RB1*-loss ROs.

Next, a small molecule named TAI-1, which potentially induces NEK2 degradation, was used to evaluate its therapeutic efficacy against Rb [36]. TAI-1 exhibited non-toxicity to ARPE-19 cells at concentrations ranging from 75 to 300 nM (Fig. S16A), but reduced the viability of Rb cell lines (Fig. 7J) while decreasing NEK2 protein levels (Fig. S16B). It induced S-phase arrest and apoptosis in Rb cells (Fig. S16C). In *RB1*^−/−^ ROs, 24-h treatment with TAI-1 (300 nM) significantly reduced cell proliferation in both early- (day 50) and middle-stage (day 80) ROs (Figs. 7K, L, S16D, E). This led to inhibition of nRPC proliferation in early stage and a reduction in the ratio of proliferative CPs in middle stages (Figs. 7M, S16F). These results demonstrate that TAI-1 has the potential to inhibit ectopic proliferation of Rb tumor cells. Overall, these findings highlight the utility of RO-derived Rb models in identifying key genes involved in Rb development.

## Discussion

In this study, we generated *RB1*-inactivated ROs across diverse genetic backgrounds to investigate pRB’s functional role and the cellular origin in Rb tumorigenesis. The *RB1*^−/−^ ROs developed Rb-like tumors both in vitro and in vivo, mimicking features of human Rb and forming serial orthotopic xenografts, whereas the *RB1*^+/-^ ROs with low pRB expression exhibited only a retinocytoma-like phenotype. Our findings demonstrate that both biallelic and monoallelic *RB1* inactivation induce overproliferation of ATOH7^+^ nRPCs, generating ectopic dividing early-born retinal cells (RGCs and CPs); however, only the former can sustainably induce overproliferation of ATOH7^+^/RXRγ^+^ nascent CPs, drives cone-rich Rb development. These insights, for the first time, reveal that nascent CPs represent the earliest cellular origin of human Rb. Additionally, we identified a potential therapeutic target for Rb through multi-omics analysis.

Xenograft models are essential for confirming the tumorigenic potential of RO-derived cells [37, 38]. While several ROs-based Rb models have been developed, only Norrie et al. and Liu et al. explored in vivo tumorigenicity [18, 19]. Our data using *RB1*-deficient ROs at day 70 also support their findings. However, Norrie et al. reported that tumors were detected 1 year post-xenograft, whereas the latency periods in Liu et al. and ours were about 3 months. Because most of the cells in ROs at day 40 are RPCs, that may not be directly affected by *RB1* loss, an in vitro model closely resembling human retinal development is superior for studying Rb initiation. Furthermore, this is the first observation of malignant transformation via serial xenograft of ROs-derived cells, underscoring the critical role of the microenvironment in tumor progression [39, 40].

While reports suggest that ARR3^+^ maturing CPs are the cell-of-origin of Rb, the *RB1* loss also promotes early CP proliferation [21], raising the possibility that tumorigenesis may originate earlier. Here, we identified the ATOH7^+^ nascent CP as an early-stage cell population in ROs that is sensitive to *RB1* loss. scRNA-seq data revealed that this cell type may directly give rise to proliferative Rb cells. Because *RB1*-null ATOH7^+^ RGCs also gain proliferative capacity during early retinogenesis [41], genes upregulated at the nRPC-stage may be another factor that activates the proliferation potential in CPs following pRB loss. However, these cells are only detectable in *RB1*-null ROs before day 70, making their identification in clinical samples challenging. Our findings suggest that the tumor initiation correlates with the timing and the cell type in which the “second hit” occurs: If the “second hit” happens before the emergence of ATOH7^+^ CPs, the tumorigenesis originates from the nascent CPs [32]. Otherwise, it originates from maturing CPs.

To date, little is known about the link between germline *RB1* mutations and the likelihood of developing Rb or retinocytoma [42]. One potential mechanism is that the first hit reduced or inactivated the expression of normal pRB [43]. In ROs with low pRB expression, Ki67^+^ cells were evaluated only in ATOH7^+^ cells but not in RXRγ^+^ or HuD^+^ cells, confirming that nRPCs are more sensitive to pRB loss. Since nRPC overproliferation generates more non-proliferative CPs and forms retinal lesions, which are considered as a retinocytoma phenotype. These results identify a retinal cell type that is more sensitive to pRB depletion than CPs, and provide the first mechanistic insights into how germline *RB1* mutations lead to retinocytoma.

There is an urgent need for new therapies to preserve vision and eyeballs in patients with Rb [44]. Recent studies have explored potential therapeutic targets using patient-derived tumors [37, 45–48]. We generated a multi-omics dataset to identify a potential therapeutic target for Rb and identified NEK2 as such a candidate. NEK2, a member of the never-in-mitosis A (NIMA) protein family, is a conserved centrosome kinase critical for cell cycle progression and differentiation [32]. NEK2 overexpression has been identified in a wide range of human cancers and enhances malignancy through multiple pathways [35, 49, 50]. Analysis of public datasets confirmed NEK2 overexpression in clinical samples. Additionally, we found that *NEK2*-targeting shRNA and TAI-1 treatments inhibited the ectopic tumor cell growth in Rb cell line and ROs. Our results comprehensively characterize the role of *NEK2* in Rb tumorigenesis and identify a druggable target for Rb and other cancers with NEK2 overexpression.

In conclusion, we successfully developed an Rb model using *RB1*-knockout ROs. Complete *RB1* loss initially induced the proliferation of ATOH7^+^ nRPCs during early retinogenesis, leading to the generation of proliferative nascent CPs that drove Rb tumorigenesis. Death-resistant CPs formed tumor tissue expressing mature cone markers. In *RB1*^+/-^ ROs with pRB downregulation, although abnormal proliferation of ATOH7^+^ nRPCs was also observed, these cells failed to produce proliferative nascent CPs but generated more normal nonproliferative CPs, forming retinocytoma lesions. Multi-omics analysis of pRB-null ROs identified a potential therapeutic target. Collectively, our findings underscore that pRB is a key factor in regulating development of Rb and retinocytoma. This study not only reveals a novel cellular origin of Rb, but also provides a foundation for developing more effective therapies.

## Materials and methods

### Ethics approval and consent to participate

The generation of hiPSCs from a retinocytoma patient was complied with the Guidance on Sample Collection of Human Genetic Diseases by the Ministry of Public Health of China and approved by Institutional Review Board of PUMCH (No. JS-2059). The patient signed an informed consent form to be included in the study. A few of human retinoblastoma samples confirmed by pathologic diagnosis were obtained from the Ocular Pathology Department at Zhongshan Ophthalmic Center (ZOC), Sun Yat-sen University (SYSU), China. NOD/SCID mice were used and approved by the Animal Experimental Ethics Committee of the ZOC, SYSU (O2021013). All methods were performed in accordance with the relevant guidelines and regulations.

### Maintenance of cell lines

All cells were maintained in a 37 °C incubator with 5% CO_2_. Y79 and Weri-Rb-1 retinoblastoma cell lines were cultured with RPMI Medium 1640 (Gibco, #C11875500BT) supplemented with 20% fetal bovine serum (FBS) (Gibco) and 1% penicillin-streptomycin (Gibco, #15140-122). ARPE19 cell line was cultured with DMEM/F12 (Gibco, #C11330500BT) with 10% FBS and 1% penicillin-streptomycin. The medium was changed every second day.

### Maintenance of hiPSCs

BC1 hiPSC was a gift from Professor Linzhao Cheng of University of Science and Technology of China [51]. Gibco hiPSC was purchased from Thermo Fisher Scientific. hiPSCs were cultured according to the previous method [15, 25]. Patient-derived hiPSC was generated by reprogramming of somatic cells from a retinocytoma patient [29]. All hiPSCs used in this study including gene-edited hiPSCs were cultured on six-well plates coated with Matrigel (Corning, #354277) in mTeSR1 medium (Stem Cell Technologies, #85850) at 5% CO_2_ in a 37 °C incubator. Medium was refreshed every second day. When it grew to 80% confluency, hiPSCs were treated with 0.5 mM EDTA (Invitrogen) for passage.

### ROs differentiation

Retinal differentiation was performed according to previous protocols [15]. Briefly, hiPSCs were digested into small clumps and cultured in suspension with mTeSR1 supplemented with 10 μM Blebbistatin (Sigma-Aldrich) to form embryoid bodies (EBs) on day 0. Then culture medium was switched into 3:1 ratio of mTeSR1/Neural induction medium (NIM) which containing DMEM/F12, 1% N2 supplement (Invitrogen), 1% non-essential amino acids (NEAA) (Gibco), 2 μg/mL heparin (Sigma-Aldrich) on day 1, 1:1 ratio of mTeSR1/NIM on day 2 and 100% NIM on day 3. EBs were plated onto a Matrigel-coated dish between day 5 and day 7. On day 16, the culture medium was changed to retinal differentiation medium (RDM) containing 72% of DMEM Basic (Gibco, #C11995500BT), 24% of DMEM/F12, 2% B27 supplement without vitamin A (Gibco), 1% NEAA and 1% antibiotic-antimycotic (Gibco) (Calculated by volume). From week 4 to week 6, neural retina domains in good condition were manually detached with a sharpened Tungsten needle and transferred to suspension culture for further induction of ROs. For long-time culture, ROs were cultured with retinal culture medium (RCM) containing RDM, 10% FBS, 100 μM Taurine (Sigma) and 2 mM GlutaMax (Gibco) within 1 week after detachment. From week 13 onwards, the medium was switched to RCM2, which B27 supplement was replaced by N2 supplement in RCM. Medium was changed every 3 days.

### Xenografts and in vivo imaging

The experimental procedures were conducted in accordance with the Association for Research in Vision and Ophthalmology Statement for the Use of Animals in Ophthalmic and Vision Research and were approved by the Animal Experimental Ethics Committee of the ZOC, SYSU. For the first round of transplantation, ROs between day 60 and day 70 were dissociated into single cells as previous reports using papain dissociation system (Worthington Biochemical) [25], and the cells were temporarily centrifuged and resuspended in DMEM/Basic medium. Prior to cell injection, 4–6 weeks old NOD/SCID mice were anaesthetized with intraperitoneal sodium pentobarbital injection (50 mg/kg, Sigma), followed by topical administration of 2.5% phenylephrine hydrochloride (for pupil dilation) and 0.5% proparacaine hydrochloride (local anesthetic). The cells were then injected into the subretinal space or vitreous cavity with 1.5 µL suspension (containing 1.0–2.0 × 10^5^ cells) per eye using a 2.5 µL microinjector and 35-gauge syringe needle (Hamilton, Switzerland). Tobramycin was used to prevent ocular infection. Spectral domain optical coherence tomography (SD-OCT) with an optical coherence tomography scanner (Heidelberg Engineering, Spectralis OCT, Germany) and anterior chamber photography with a slit lamp digital imaging system (Topcon, SL-D7/DC-3/IMAGEnet, Japan) were used to track and monitor the grafts. After 13 weeks of injection, the mice were euthanized and tumor-bearing eyes were used for further analysis. In the second round of xenograft, the tumor cells that from first xenograft were dissociated into single cells with 0.5 mM EDTA for 5 min and then 1.0–2.0 × 10^5^ cells for each eye were used for transplantation. The procedure for the second round of xenograft followed the same protocol as the first round, with tumor cells collected and analyzed after 13–15 weeks.

### Teratoma formation and analysis

The experimental procedures were approved by the Animal Experimental Ethics Committee of the ZOC, SYUS. Experiment was performed as our previous reports. 1–1.5 × 10^6^ hiPSCs with 30% Matrigel were injected into the nuchal region of NOD/SCID mice. After 6–8 weeks post-injection, the mice were humanely euthanized and teratomas were surgically excised, fixed in formalin, embedded in paraffin, sectioned and stained with H&E. Images were taken using a Zeiss Axio Scan. Z1 Slide Scanner (Carl Zeiss, Jena, Germany).

### Targeting, donor plasmid construction

Two *RB1*^−/−^ hiPSC lines were generated via CRISPR/Cas9-mediated gene editing, using human BC1 and Gibco hiPSC as parental lines. For the BC1 knockout cell line, a single guide RNA (sgRNA) was designed by the online website: http://crispor.tefor.net/ (5’-GGTGGCGGCCGTTTTTCGGG-3’), which targets the Coding Sequence (CDS) of *RB1* exon 1, and inserted into pSpCas9(BB)-2A-Puro (PX459 V2.0, Addgene) vector to generate the targeting plasmid PX459-RB1-sgRNA. Similarly, the same sgRNA was inserted into pSpCas9(BB)-2A-GFP (PX458, Addgene) vector to establish the targeting plasmid PX458-RB1-sgRNA. hPGK-Puro-polyA resistance cassette was flanked by left and right homologous arms. The above fragment was then inserted into pMD19-T (Takara, #D102A) vector to generate the donor plasmid pMD19-LA-Puro-RA.

### Cell transfection and resistance selection

Electroporation was carried out as previously described [25]. In brief, hiPSCs were dissociated into single cells using Accutase (Gibco, #A1110501) at 37 °C for 5 min, Approximately 15 µg PX459-RB1-sgRNA plasmid for BC1 hiPSCs or 10 µg PX458-RB1-sgRNA and 10 µg pMD19-LA-Puro-RA plasmid for Gibco hiPSCs were used for further nucleofection. After 3 days of transfection, resistant single cell clones were selected with puromycin (Solarbio) at a concentration of 0.4 µg/mL for BC1-*RB1*^−/−^ hiPSCs or 1 µg/mL for Gibco-*RB1*^Puro/Puro^ hiPSCs. These clones were amplified and used for further validation.

### Mutation analysis

Genomic DNA was extracted using an Animal Genomic DNA Rapid Extraction Kit (Beyotime). PCR was performed using primers (primer VF1, VR1 for BC1-*RB1*^−/−^ hiPSC, VF2, VR2, VF3, VR3 for Gibco-*RB1*^Puro/Puro^ hiPSCs and Gibco-*RB1*^w/Puro^ hiPSCs, see sequence details in Table S3) depending on which exon was mutated in the sample. The mutation was verified by Sanger sequencing.

### RT-PCR analysis

Total RNA was extracted from hiPSCs using Trizol Reagent (Sigma). Then, 1 μg of total RNA was reverse-transcribed using the PrimeScript™ RT reagent Kit (Takara, #RR047A) to synthesize cDNA. The VF4/VR4 primer pair was used for PCR to verify the mutant effect (see sequence details in Table S3). The resulting PCR products were used for further Sanger sequencing.

### Karyotype analysis

Karyotype analysis was performed using G-band staining. hiPSCs were treated with 20 µg/mL colchicine for 1 h. Cells were then dissociated with 0.5 mM EDTA for 5 min, resuspended and incubated in 0.075 M KCl solution for 20 min, and then fixed with a 3:1 methanol/acetic acid mixture for 10 min. After dropping onto cold slides for 2 h, chromosomes were observed under an OLYMPUS BX43 Microscope and analyzed with applied spectral imaging system.

### Cell cycle analysis

Cell cycle profiles were analyzed using the Cell Cycle Staining Kit (MultiScience, Hangzhou, China) according to the manufacturer’s instructions. Briefly, cells were dissociated into single cells. After washing twice with PBS, cells were fixed with cold 75% ethanol overnight. The cells were then washed, treated with RNase and stained with PI in the dark for 30 min. The percentage of cells in the sub G1, G0/G1, S and G2/M phases of the cell cycle was analyzed using a BD LSR Fortessa cytometer (BD Biosciences).

### Immunocytochemistry

For cryosection analysis, samples were fixed in 4% paraformaldehyde (PFA) for 30 min and dehydrated in a gradient of sucrose solutions. After embedding in OCT compound (Sakura Finetek Japan, Tokyo, Japan), tissues were sectioned at a thickness of 12–16 μm. For flat-mounts analysis, samples were fixed in 4% PFA for 15 min. Then samples were blocked and permeabilized in 0.4% TritonX-100 with 1% Donkey serum for 1 h at room temperature and then incubated with primary antibody at 4 °C overnight. The following day, samples were rinsed with PBS and subsequently incubated with secondary antibodies for 1 h at room temperature in the dark. Nuclei were stained with DAPI solution (Dojindo). Images were obtained using the Zeiss LSM 880 confocal microscope (Carl Zeiss). Antibodies information is detailed in Table S4. Quantification of positive cells was performed using ImageJ or QuPath software [52,53]. Quantification of retinal dyslamination was performed according to a recent report [54].

### Hematoxylin and Eosin staining

Human Rb samples and ROs were fixed in 10% neutral buffered formalin, embedded in paraffin, sectioned and stained with hematoxylin (Biosharp, Hefei, China) and eosin (Beyotime Biotechnology, Shanghai, China) (H&E). Images were taken using a Zeiss Axio Scan. Z1 (Carl Zeiss, Jena, Germany).

### EdU labeling

EdU labelling was performed using Click-iT EdU Imaging Kits (Invitrogen). ROs at the indicated time were cultured in RCM in the presence of 10 μM EdU for 24 h before collecting. After fixation, dehydration, embedding, and sectioning, EdU was detected by Click-iT assay according to the manufacturer’s protocol.

### CCK8 assay

CCK8 (Cell Counting Kit-8) colorimetric assays were performed to detect the cell viability in ARPE19, Y79 and Weri-Rb-1 cells according to the manufacturer’s method (GLPBIO). Briefly, approximately 1 × 10^4^ cells were seeded in 96-well plate and incubated with or without different concentrations of TAI-1 (ApexBio). After 72 h, CCK8 reagent was applied to each well and incubated for 2 h at 37 °C. The absorbance at 450 nm of each well was measured using a multimode microplate reader (Synergy H1; BioTek) to calculate the relative cell viability.

### Western blot

hiPSCs, retinoblastoma cell lines, ARPE19 cell line or ROs were harvested and washed with 1×PBS, with the addition of cold RIPA lysis buffer (Beyotime, #P0013B) and 1×PMSF (Beyotime, #ST507), followed by incubation on ice for 30 min in a shaker. The lysate supernatant was retained after 15 min centrifugation (4 °C) for SDS-PAGE. The BCA Protein Assay Kit (Beyotime) was used to determine the protein concentration of each sample. Approximately 10 to 20 µg of proteins from cells or organoids lysates were mixed with Loading Buffer (Beyotime, #P0015L), incubated at 95–100 °C for 10 min and separated on 10% Bis-Tris gel in Tris-Glycine Running Buffer (Sangon, #C520001). The proteins were transferred to PVDF membranes (Millipore, #HVLP02500) with a 45 μm pore size using Apparatus (Bio-Rad). Membranes were blocked with 5% non-fat milk solution or 5% BSA for 1 h, then incubated with primary antibodies (see Table S4 for details) at 4 °C overnight, followed by incubation with secondary antibodies for 1 h at room temperature using horseradish peroxidase-conjugated anti-rabbit or anti-mouse IgG. Signals were generated using Immobilon Western Chemilum HRP Substrate (Millipore, #WBKLS0100) and visualized by Bio-Rad ChemiDoc Touch system or Tanon 5200.

### Transmission electron microscope (TEM)

For TEM analysis, ROs and tissues were dehydrated by passing through a graded series of ethanol solutions and embedded in epoxy resin. Samples were then cut into ultrathin sections with an ultra-microtome, stained with 1% uranyl acetate and lead citrate, and then imaged with an electron microscope (FEI Europe, Eindhoven, the Netherlands).

### Bulk RNA-seq analysis

ROs or hiPSCs were collected for RNA extraction. Total RNA was isolated with Trizol reagent (Invitrogen) according to the manufacturer’s method. RNA quality was assessed using Agilent 2100 Bioanalyzer (Agilent Technologies). mRNA was enriched using Oligo(dT) beads and then broken into short fragments. The fragments were further reverse transcribed into cDNA using NEBNext Ultra RNA Library Prep Kit for Illumina (New England Biolabs). The cDNA was further repaired at the ends and amplified by PCR to generate a sequencing library. RNA sequencing was performed by Illumina Novaseq6000 Sequencer.

Raw data were processed using the fastp tool (version 0.18.0) [55]. Reads containing poly-Ns, duplicate sequences, and low-quality sequences were removed to obtain high-quality clean reads. The remaining clean reads were further used in assembly and gene abundance calculations. Gene expression levels were quantified by RSEM software (version 16). The fragment per kilobase transcript per million mapped reads (FPKM) value was calculated to quantify the expression levels of each gene. Correlation analysis was performed by R. correlation between two parallel experiments. Principal Component Analysis (PCA) was performed with R package g models (http://www.r-project.org/). Differential expression analysis was performed by DESeq2 R package (1.18.0) between two different groups and edgeR between two samples. Genes/transcripts with the false discovery rate (FDR) parameter below 0.05 and absolute fold change ≥2 were considered DEGs (different expression genes). Then, the DEGs were analyzed by Gene Ontology (GO) and Kyoto Encyclopedia of Genes and Genomes (KEGG) pathway enrichment. GO terms or pathways with a *Q* value ≤ 0.05 were defined as significantly enriched. Gene set enrichment analysis (GSEA) was further performed using GSEA and MSigDB software. Enrichment scores and *p* values were calculated in default parameters, and *P* < 0.05 was considered statistically significant.

### ATAC-seq analysis

Nuclei suspensions were incubated in a transposition reaction Mix containing Tn5 transposase. which preferentially fragments accessible chromatin regions while simultaneously adding adapter sequences to the DNA ends. The reaction was carried out at 37 °C for 30 min, followed by purification using the QIAGEN MinElute PCR Purification Kit. The resulting DNA fragments were then amplified and subjected to paired-end sequencing on the Illumina NovaSeq 6000 platform.

After sequencing, raw reads were processed to remove low-quality sequences, and the clean reads were aligned to the reference genome using Bowtie2 [56], peak scanning was performed by MACS2 [57]. The read-enriched regions would be defined as a peak when log2 (fold enrichment above background) ≥3 and –log10 (*P*-value) ≥ 3. After that, the peak-related genes were identified according to the genomic location information and gene annotation information of peak, peak-related genes can be confirmed using ChIPseeker [58]. Pathway enrichment analysis identified significantly enriched metabolic pathways or signal transduction pathways in peak-related genes compared with the whole genome background.

### Single-cell RNA-seq analysis

#### Library preparation and sequencing

At day 80, a total of 15–20 ROs were dissociated into single cells using papain enzyme according to the manufacturer’s instructions (Worthington Biochemical). Cell suspension was mixed with an equal volume of 0.4% trypan blue and the cells were counted using a Countess® II Automated Cell Counter to adjust the concentration of live cells to the desired concentration (1000–2000 cells/μL). The cell suspension was then loaded onto a 10x Genomics GemCode Single-cell instrument to generate GEMs (Gel Beads-In-Emulsions). The libraries were prepared using Chromium Next GEM Single Cell 3’ Reagent Kits version 3.1 and sequenced in PE150 mode at an Illumina Novoseq6000 platform. A total of 9085 cells in WT ROs and 9520 cells in C5 *RB1*^−/−^ ROs were generated respectively.

#### Cell clustering

CellRanger (version 3.1.0) software was used for quality control and expression quantification. Samples were de-multiplexed and aligned to the human reference genome GRCh38 to calculate the cell-gene expression matrix.

The gene expression matrices of cells were submitted to Seurat (version 3.1.1) for further analysis [59, 60]. Cells with unusually high numbers of UMIs (Unique Molecular Identifier) ≥ 8000 or mitochondrial gene percent ≥10% were filtered out. Cells with less than 500 or more than 6000 genes detected were also excluded. Additionally, doublet GEMs were also filtered out by using DoubletFinder (v2.0.3) [61]. Seurat software’s LogNormalize method was used to normalize gene expression, FindVariableFeatures method was used to find intercellular high signature genes. Samples were then integrated using Canonical Correspondence Analysis (CCA) correction to remove batch effects. Finally, the data were normalized to Z-score using the Seurat’s ScaleData function, Cell clustering was performed by Louvain method with maximum modularity.

#### Cell cycle analysis

The Seurat was used to assign the cell cycle score to each cell from 15 G1 phase genes, 20 S phase genes, 61 G2 phase genes and 64 M phase genes based on the previous reports[62].

#### Pseudotime trajectory analysis

Single-cell trajectory was analyzed using matrix of cellular gene expression matrices by Monocle 2 (Version 2.6.4) [63]. The high-dimensional cell expression data were projected into a two-dimensional space by a dimensionality reduction method (DDRTree) to order the cells (sigma = 0.001, lambda = NULL, param.gamma = 10, tol = 0.001), DEGs across different clusters (FDR<1e-5) were used to reconstruct the biological trajectory tree based on pseudo time.

#### Online data sources and bioinformatic analysis

Three microarray or gene expression dataset (GSE97508, GSE24673, GSE111168) were retrieved from Gene Expression Omnibus (GEO) database (https://www.ncbi.nlm.nih.gov/geo/). Totally 12 retinoblastoma samples and 8 normal retina samples were obtained from these three data sets.

The common DEGs from three datasets (RNA-seq, scRNA-seq and ATAC-seq) were analyzed by the Search Tool for the Retrieval of Interacting Genes (STRING) database to investigate the interaction (https://www.string-db.org) [64]. To identify the hub genes in this network, the data from STRING were imported into the CytoHubba program (Cytoscape, https:// https://cytoscape.org). The top 10 nodes withthe highest degrees were selected as hub genes.

#### NEK2-targeting Lentiviral transduction of Y79 cells and ROs

Lentiviruses encoding Scramble (SCR) or NEK2 shRNA were synthesized by VectorBuilder (Guangzhou, China). The shRNA sequences were designed based on previous reports (see sequence details in Table S3). For transduction of Y79, shNEK2 and SCR shRNA lentivirus were added to cells at a multiplicity of infection (MOI) of 10. Following 12 h of incubation, the viral supernatant was aspirated and replaced with fresh medium. Cells were cultured for an additional 60 h before harvesting for subsequent Western blot and CCK8 cell viability assays. For transduction of ROs, lentivirus-polybrene mixtures (5 μg/mL final concentration) were prepared in 50 μL RC2 medium and applied to ROs at an MOI of 40. After 24 h of incubation, the viral supernatant was removed and replaced with fresh RC2 medium. ROs were maintained for 72 additional hours before processing for immunofluorescence analyses.

### Statistical analysis

All experiments were repeated at least three times. Values were expressed as mean ± standard deviation (SD). Statistical analysis was performed with GraphPad Prism version 9.0. The statistical significance of difference was determined by unpaired *t* test or one-way ANOVA followed by Dunnett’s test and *P* value below 0.05 was considered statistically significant.

## Supplementary information

Supplemental information

Original data

## Data Availability

The RNA-seq, scRNA-seq and ATAC-seq data have been deposited in the Genome Sequence Archive (Genomics, Proteomics & Bioinformatics 2025) in National Genomics Data Center (Nucleic Acids Res 2025), China National Center for Bioinformation/Beijing Institute of Genomics, Chinese Academy of Sciences (GSA-Human: HRA005121) that are publicly accessible at https://ngdc.cncb.ac.cn/gsa-human.
