# Supplementary material for: Longitudinal analysis of retinal cell state transitions in RB1-deficient retinal organoids reveals the nascent cone precursors are the earliest cell-origin of human retinoblastoma
Source: Cell Death Dis. 2026 Jan 14;17(1):34. doi: 10.1038/s41419-025-08191-x (PMC12805002; doi:10.1038/s41419-025-08191-x)
Supplement: Supplementary file 1 — Supplemental information [file 41419_2025_8191_MOESM1_ESM.docx]

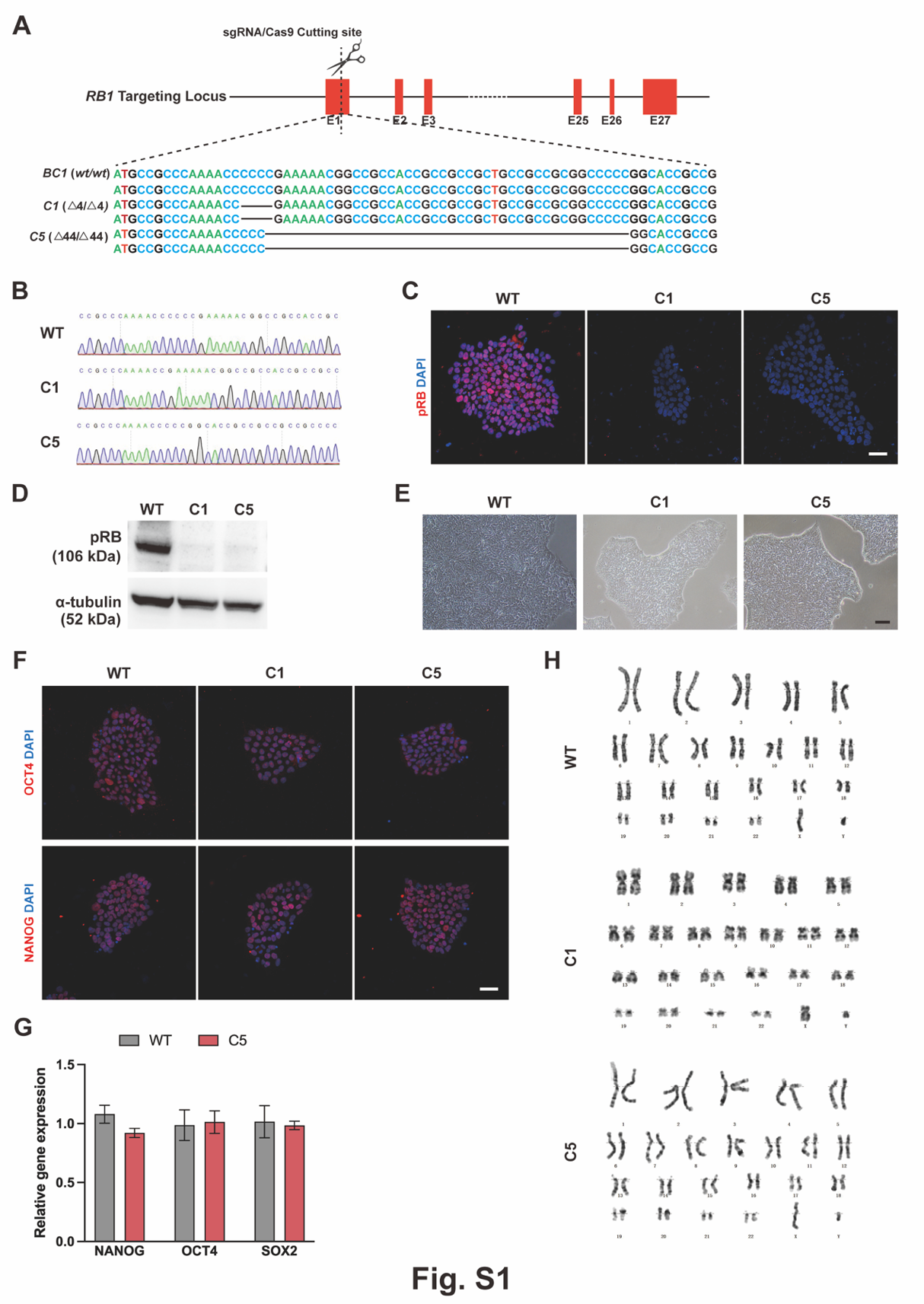


Fig. S1 Generation and characterization of *RB1*^-/-^ hiPSCs

1. Target strategy of generation of *RB1*^-/-^ hiPSCs from BC1 hiPSCs.
2. Genotypic validation of hiPSC lines by Sanger sequencing.
3. Representative immunostaining images of pRB in WT and *RB1*^-/-^ hiPSCs.
4. Western blot analysis of pRB in WT and *RB1*^-/-^ hiPSCs.
5. Representative bright field images of WT and *RB1*^-/-^ hiPSCs.
6. Representative immunostaining images of OCT4 and NANOG in wild type and *RB1*^-/-^ hiPSC lines.
7. Relative expression of pluripotency genes in WT and *RB1*^-/-^ hiPSCs, n = 3.
8. Karyotype analysis of different hiPSC lines.

Scale bars = 200μm (C, E, F)


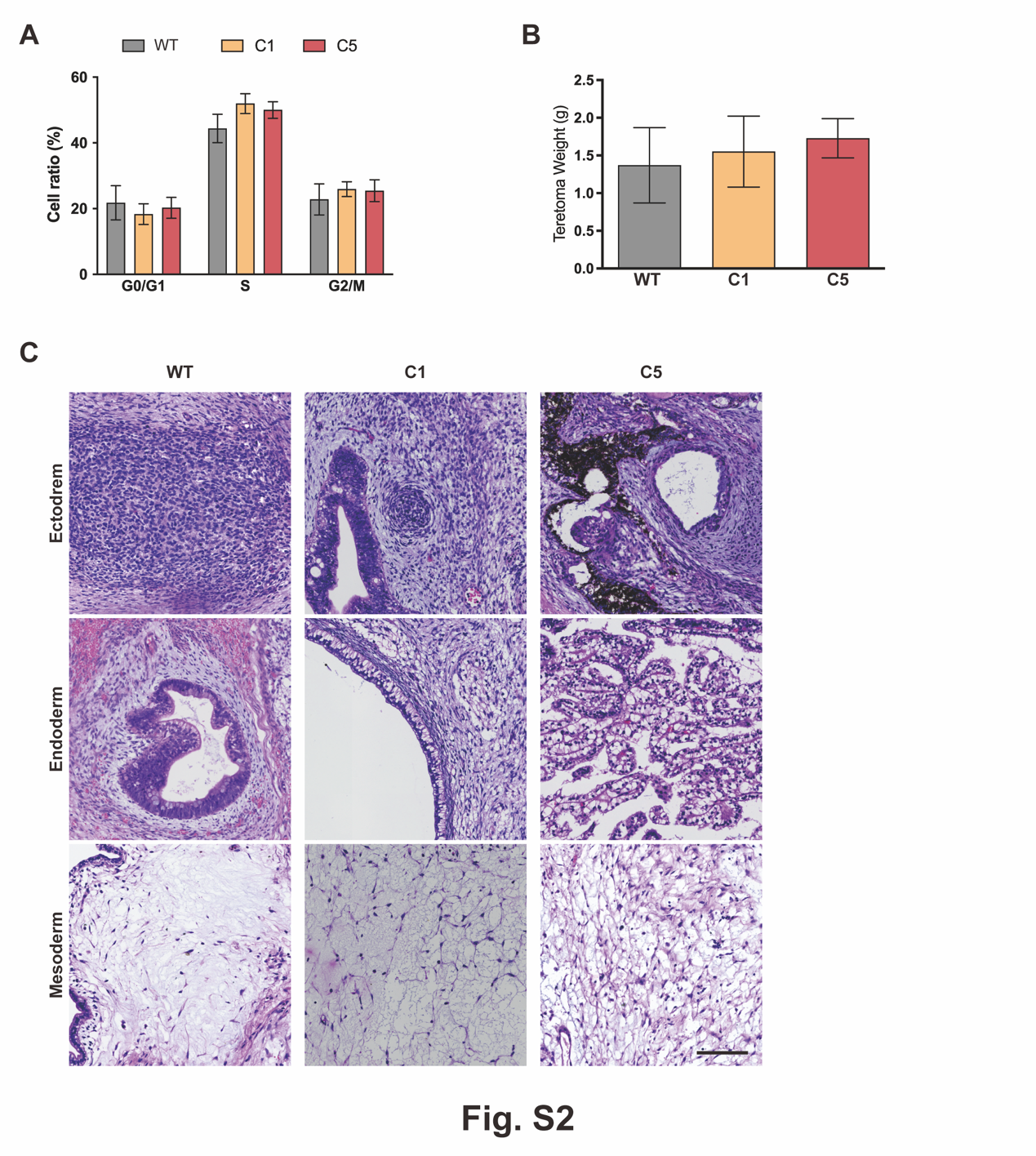


Fig.S2 Cell cycle and multiple differentiation potential of *RB1*^-/-^ hiPSCs.

1. Cell cycle distribution of WT and *RB1*^-/-^ hiPSCs, n = 5.
2. Quantification of weight of teratomas generated from WT and *RB1*^-/-^ hiPSCs, n = 5.
3. Representative H&E staining images of teratomas generated from WT and *RB1*^-/-^ hiPSCs. Scale bars = 100 μm (C)


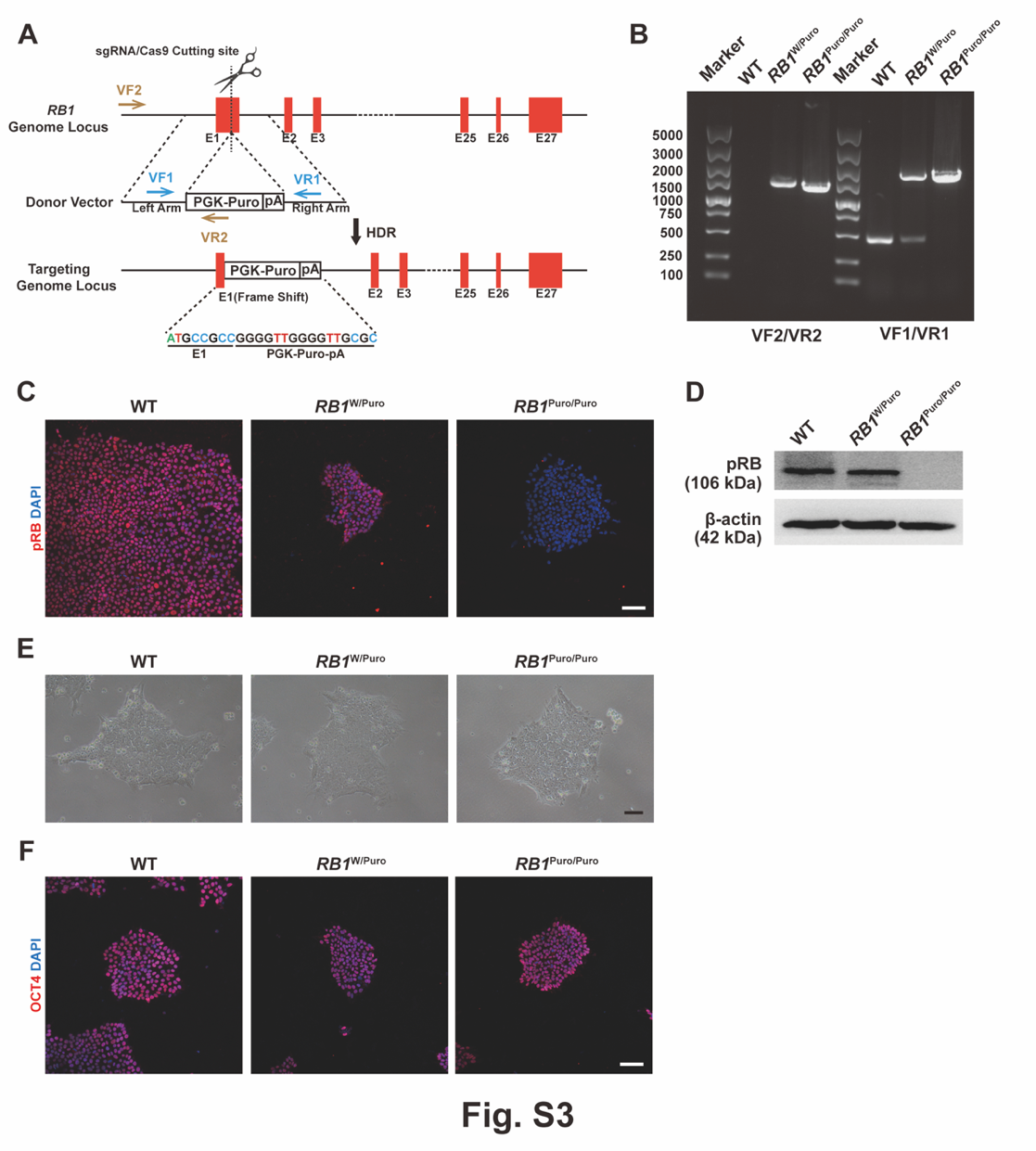


Fig. S3 Generation and characterization of Gibco line-derived *RB1*^-/-^ and *RB1*^+/-^ hiPSCs

1. Targeting strategy for generation of *RB1*^W/Puro^ and *RB1*^Puro/Puro^ hiPSC lines from Gibco hiPSCs. Two primer pairs were designed to validate the *RB1* gene knockout and insertion of PGK-puromycin cassette.
2. Genotypic characterization of gene-edited Gibco hiPSCs. Zygosity analysis using flanking primers VF1/VR1 identified heterozygous (*RB1*^w/Puro^) and homozygous (*RB1*^Puro/Puro^) hiPSC lines. Puromycin cassette integration was verified by junction PCR with VF2/VR2 primers.
3. Representative immunostaining for pRB in WT, *RB1*^W/Puro^ and *RB1*^Puro/Puro^ hiPSCs.
4. Western blot analysis of pRB in WT, *RB1*^W/Puro^ and *RB1*^Puro/Puro^ hiPSCs.
5. Representative bright field images of WT, *RB1*^W/Puro^ and *RB1*^Puro/Puro^ hiPSCs.
6. Representative immunostaining images for OCT4 in WT, *RB1*^W/Puro^ and *RB1*^Puro/Puro^ hiPSCs.

Scale bars = 100 μm (C, F) and 200 μm (E)


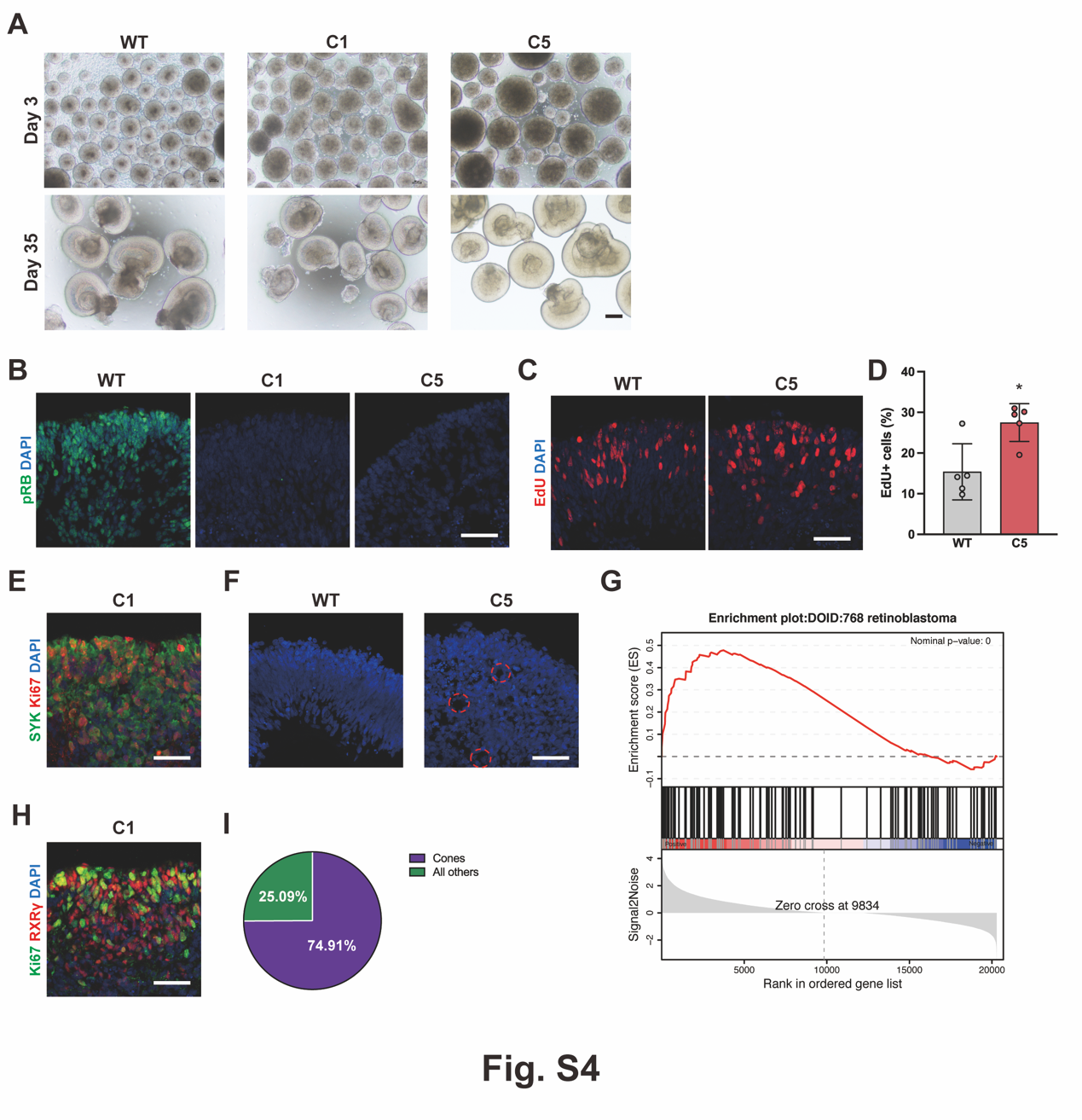


Fig. S4 Induction of ROs from *RB1*^-/-^ hiPSCs

1. Representative bright field images of EBs and early ROs in WT and *RB1*^-/-^ ROs.
2. Representative immunostaining images of pRB in WT and *RB1*^-/-^ ROs at day 50.
3. Representative EdU-labeling images in WT and *RB1*^-/-^ ROs at day 70.
4. Quantification of EdU-labeled cells at day 70. Data represents mean ± SD. * *P* < 0.05 vs. WT, n = 5.
5. Representative immunostaining of SYK and Ki67 in C1 *RB1*^-/-^ ROs at day 90.
6. Representative DAPI staining images in WT and *RB1*^-/-^ ROs at day 90. The dash lines indicate the rosette structure.
7. Up-regulation of Rb-related genes in *RB1*^-/-^ ROs by Gene set enrichment analysis (GSEA).
8. Representative immunostaining for Ki67 and RXRγ in C1 *RB1*^-/-^ ROs at day 90.
9. Quantification of RXRγ^+^ cells in C1 *RB1*^-/-^ ROs.

Scale bars = 200 μm (A) and 50 μm (B, C, E, F, H)


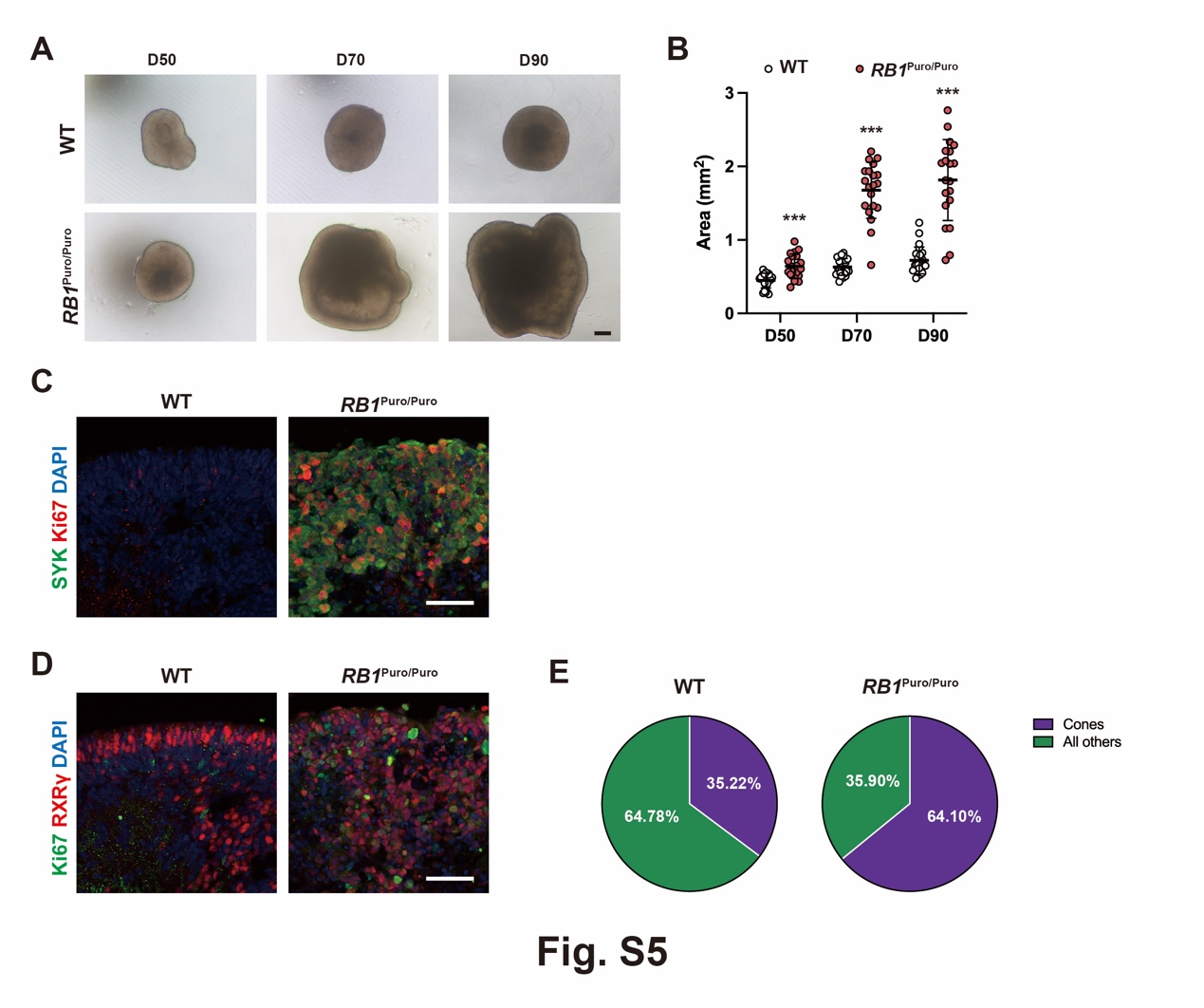


Fig. S5 Reproducibility of tumorigenesis in *RB1*^Puro/Puro^ ROs from Gibco hiPSC line

1. Representative microscopic images of WT and *RB1*^Puro/Puro^ ROs from day 50 to day 90.
2. Quantification of size of ROs in different culture phase. Data represents mean ± SD (n = 20-24). *** *P* < 0.001 vs. WT, n = 24 (WT), n=20 (*RB1*^Puro/Puro^).
3. Representative immunostaining for SYK and Ki67 in WT and *RB1*^Puro/Puro^ ROs at day 90.
4. Representative immunostaining for Ki67 and RXRγ in WT and *RB1*^Puro/Puro^ ROs at day 90.
5. Quantification of RXRγ^+^ cells in WT and *RB1*^Puro/Puro^ ROs.

Scale bars = 200 μm (A) and 50 μm (C, D)


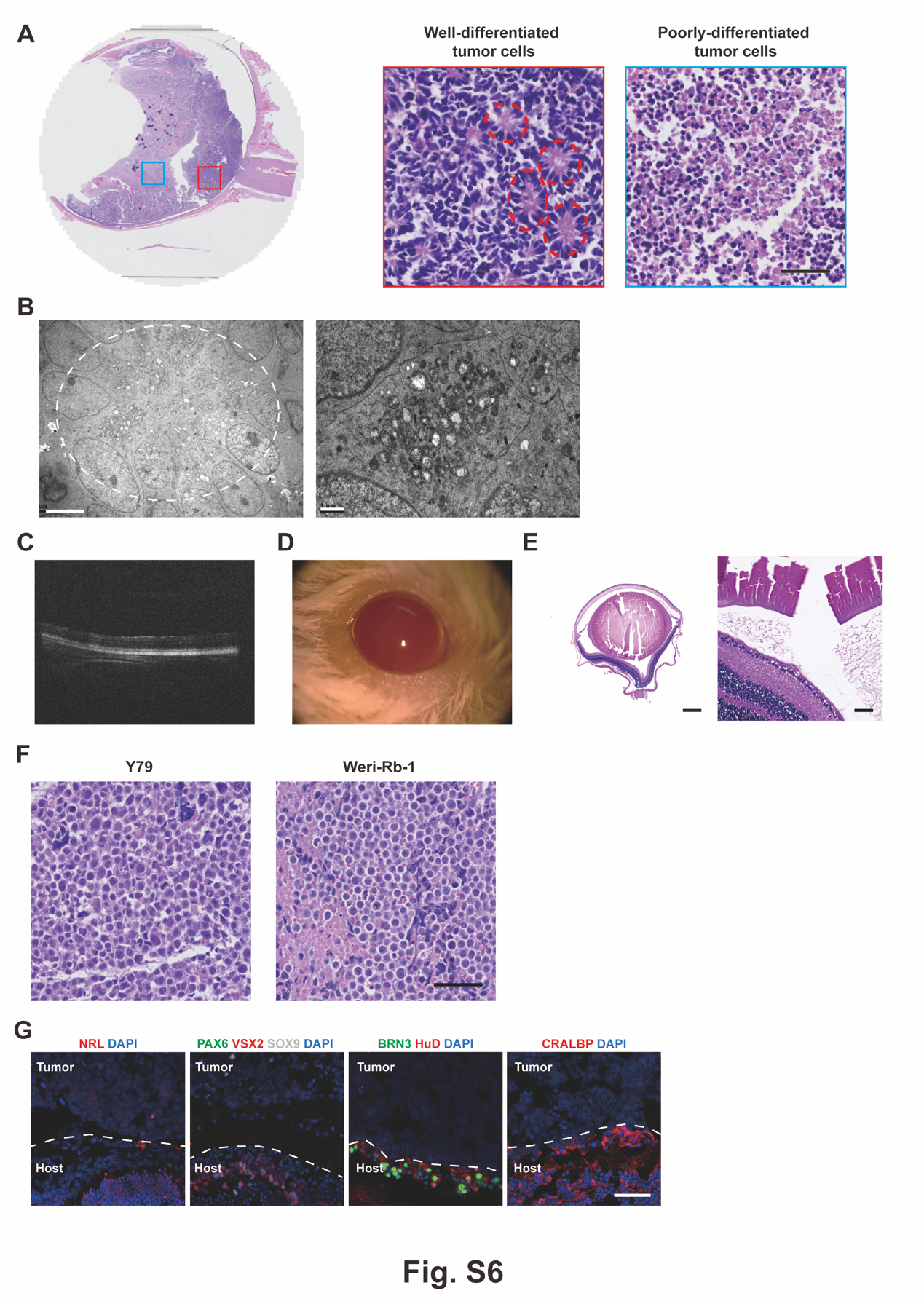


Fig. S6 Tumorigenesis of *RB1*^-/-^ ROs *in NOD-scid mice.*

1. Representative H&E staining images of Rb patient samples showing the well-differentiated and poorly-differentiated tumor cells.
2. Representative transmission electron microscopy (TEM) images of tumor cells derived from *RB1*^-/-^ ROs in 1st xenografts, showing characteristic rosette structures and abundant mitochondria. The dash line represents the Flexner-Wintersteiner rosette structure.
3. Representative OCT images of engrafted eyes at week 10 after injection of cells from WT group.
4. Representative slit-lamp images of engrafted eyes at week 13 after injection of cells from WT group. No tumor cells were observed in anterior chambers.
5. Representative H&E staining images of retina after injection of cells from WT group.
6. Representative H&E staining images of eyes with injection of Y79 or Weri-Rb-1 cells for 4 weeks.
7. Representative immunostaining for NRL, PAX6, VSX2, BRN3, HuD and CRALBP in xenograft.

Scale bars = 50 μm (A, F, G), 5 (left) and 1 (right) μm (B), 500 (left) and 50 (right) μm (E)


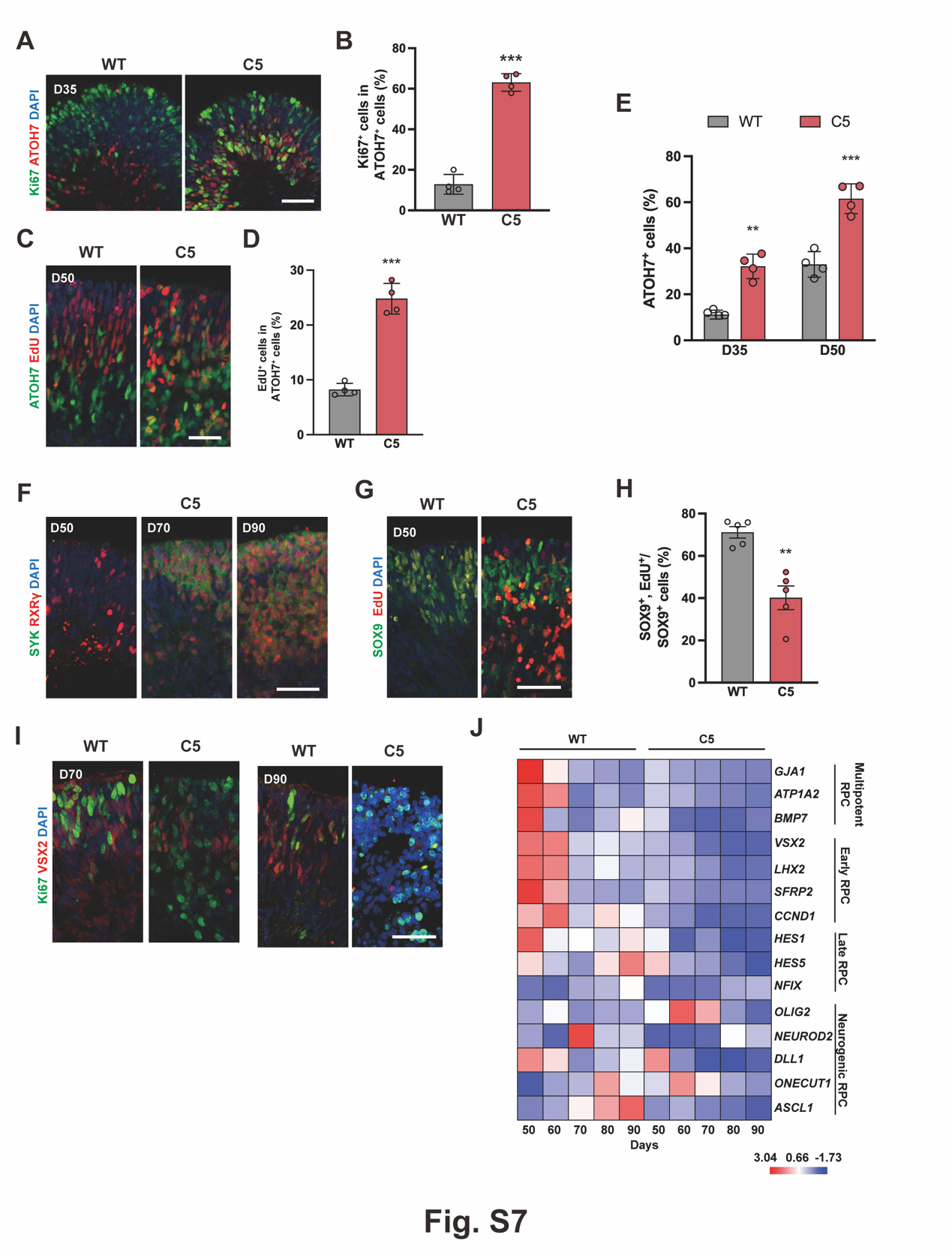


Fig. S7 Induction of cell cycle re-entry after *RB1* loss

1. Representative immunostaining for Ki67 and ATOH7 in WT and *RB1*^-/-^ ROs at day 35.
2. Quantification of Ki67^+^ cells in ATOH7^+^ cells in WT and *RB1*^-/-^ ROs at day 35. Data represents mean ± SD. *** *P* < 0.001 vs. WT, n = 4.
3. Representative immunostaining for EdU and ATOH7 in WT and *RB1*^-/-^ ROs after EdU labeling.
4. Quantification of EdU-labeled cells among ATOH7^+^ in WT and *RB1*^-/-^ ROs. Data represents mean ± SD. *** *P* < 0.001 vs. WT, n = 4.
5. Quantification of the ratio of ATOH7^+^ cells in WT and *RB1*^-/-^ ROs at day 35 and 50. ** *P* < 0.01, *** *P* < 0.001 vs. WT, n = 4.
6. Representative immunostaining for SYK and RXRγ in *RB1*^-/-^ ROs from day 50 to 90.
7. Representative immunostaining for EdU and SOX9 in WT and *RB1*^-/-^ ROs after EdU labeling.
8. Quantification of the ratio of EdU^+^/SOX9^+^ cells among SOX9^+^ cells in WT and C5 *RB1*^-/-^ ROs. Data represents mean ± SD. ** *P* < 0.01 vs. WT, n = 5.
9. Representative immunostaining for Ki67 and VSX2 in WT and *RB1*^-/-^ ROs from day 70 to 90.
10. Heatmap of differential expression of RPC marker genes in WT and *RB1*^-/-^ ROs from day 50 to 90.

Scale bars = 50 μm (A, C, F, G, I)


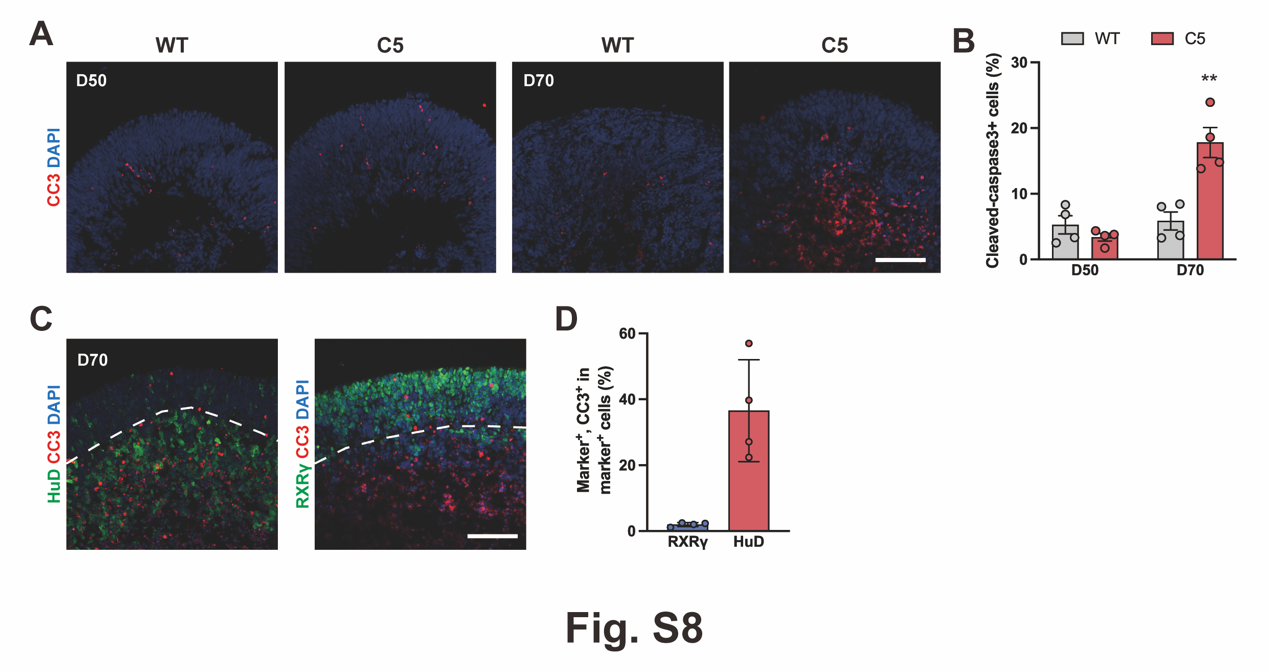


Fig. S8 Cell apoptosis in *RB1*^-/-^ ROs-derived RGCs

1. Representative immunostaining for cleaved caspase 3 (CC3) and HuD in WT and *RB1*^-/-^ ROs at day 50 and day 70
2. Quantification of CC3^+^ cells in WT and *RB1*^-/-^ ROs at day 50 and day 70. Data represents mean ± SD. ** *P* < 0.01 vs. WT, n = 4.
3. Representative immunostaining for cleaved caspase 3 (CC3), HuD and RXRγ in *RB1*^-/-^ ROs at day 70
4. Quantification of the ratio of CC3^+^ cells in RXRγ^+^ cells or HuD^+^ cells at day 70. Data represents mean ± SD, n = 4.

Scale bars = 100 μm (A, C)


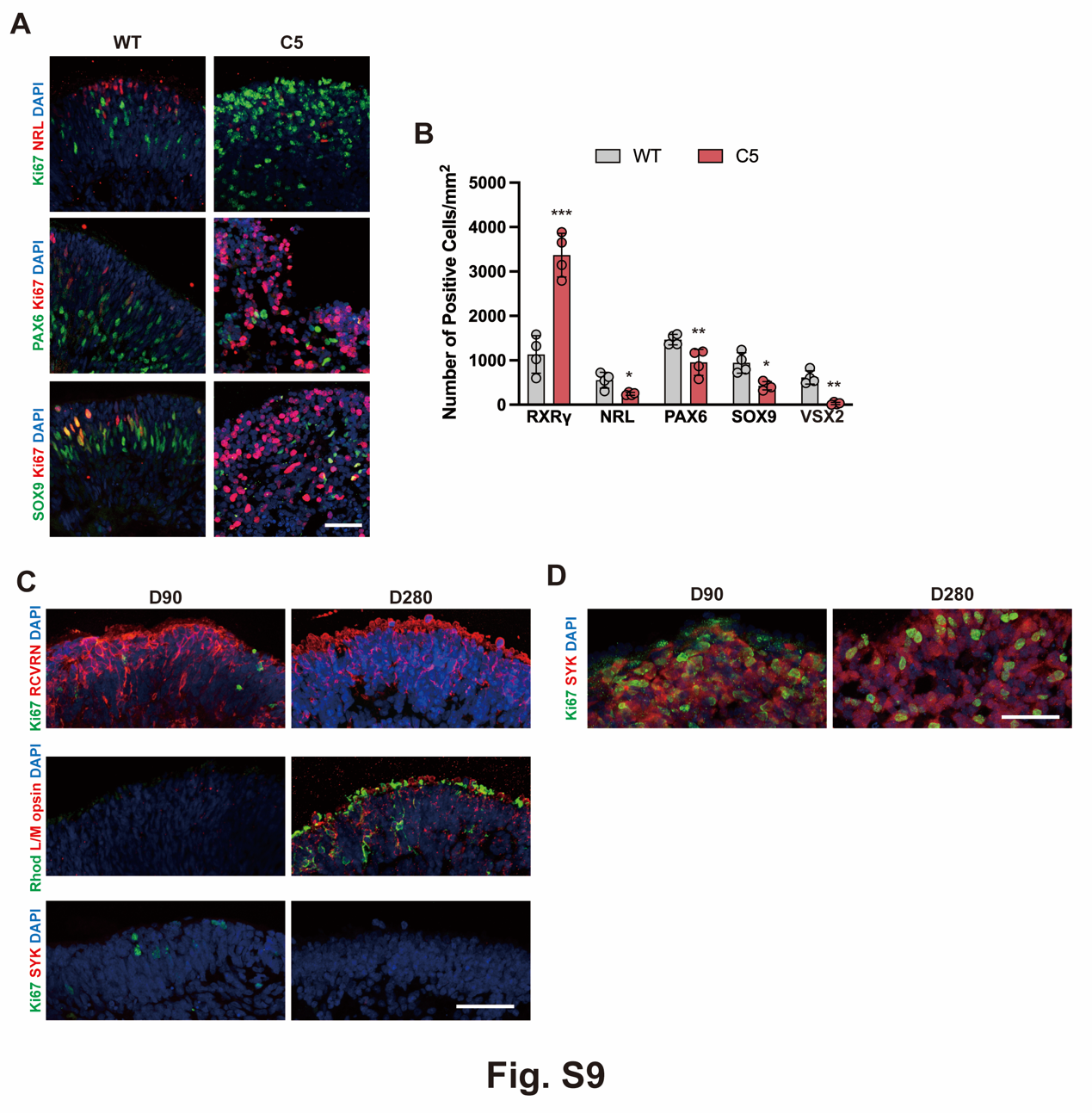


Fig. S9 Maturation of *RB1*^-/-^ ROs

1. Representative immunostaining for NRL, PAX6, SOX9 and Ki67 in WT and C5 *RB1*^-/-^ ROs at day 90.
2. Quantification of cell number of RXRγ^+^, NRL^+^, SOX9^+^, PAX6^+^ and VSX2^+^ cells in WT and *RB1*^-/-^ ROs at day 90. Data represents mean ± SD. * *P* < 0.05, ** *P* < 0.01, *** *P* < 0.001 vs. WT, n = 4.
3. Representative immunostaining for RCVRN, Ki67, Rhodopsin, L/M opsin and SYK in WT ROs at day 90 and day 280.
4. Representative immunostaining for Ki67 and SYK in *RB1*^-/-^ ROs at day 160 and day 280.

Scale bars = 50 μm (A, C, D)


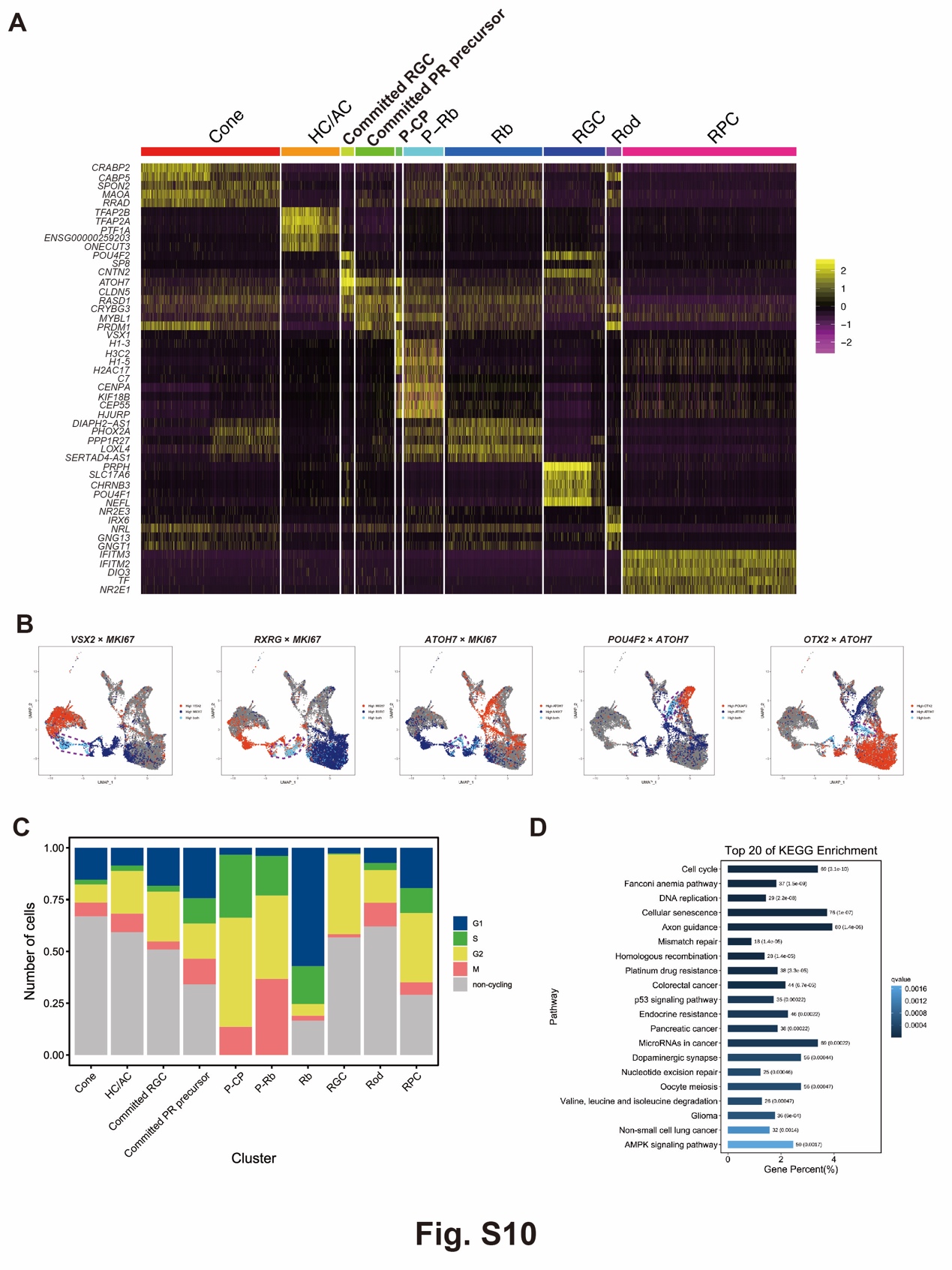


Fig. S10 scRNA-seq of WT and *RB1*^-/-^ ROs at day 80

1. Heatmap visualization of enriched genes for each cell types.
2. Feature plots of double positive cells in WT and *RB1*^-/-^ ROs.
3. Quantification for ratio of cells in each cell cycle phase.
4. KEGG enrichment for DEGs between Rb cells (Rb, P-Rb) and other retinal cell types.


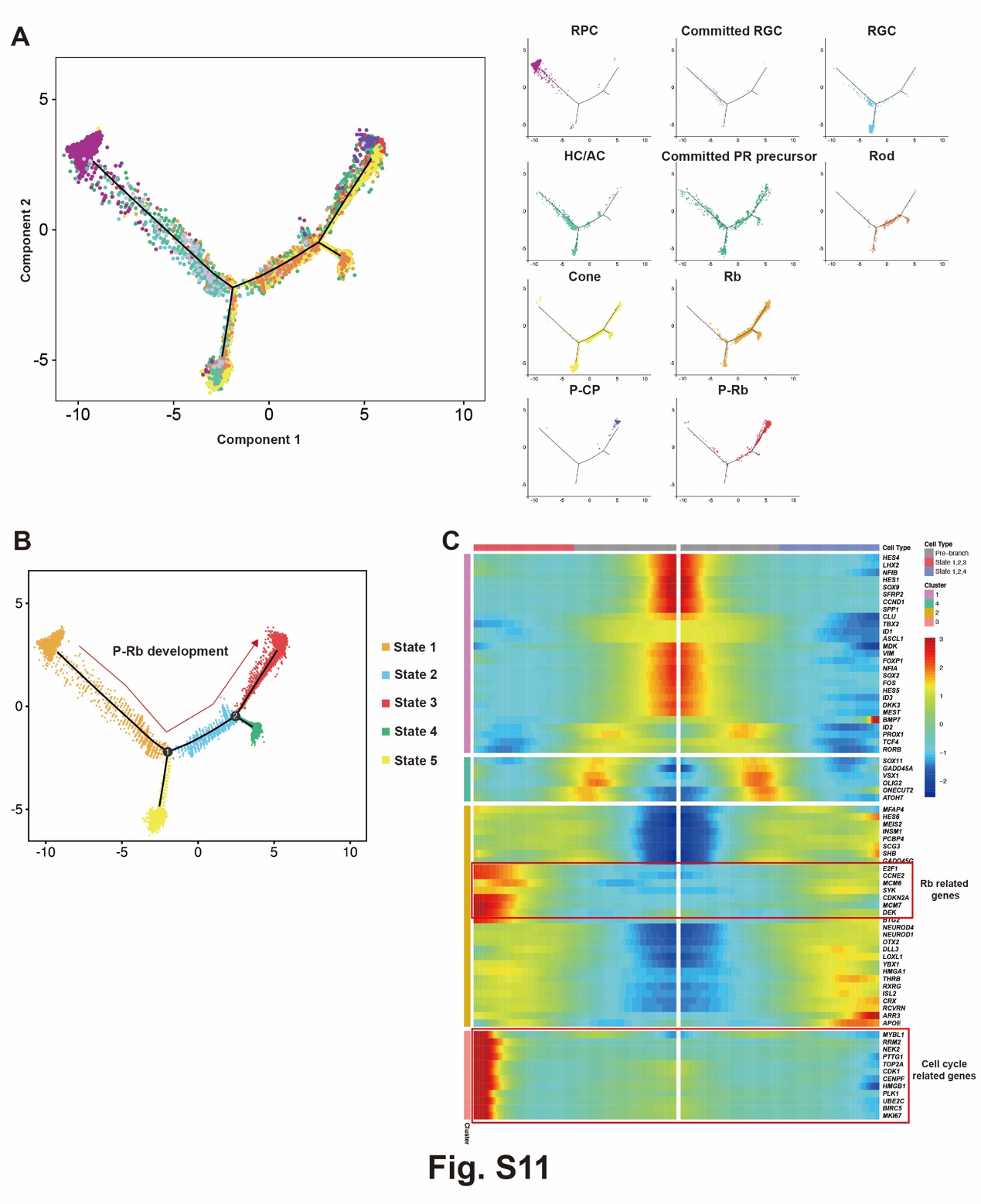


Fig. S11 Construction of Rb cells differentiation trajectory by *RB1*^-/-^ ROs

1. Trajectory analysis of *RB1*^-/-^ ROs by monocle 2 in each cell cluster.
2. Pseudo-time trajectory analysis of *RB1*^-/-^ ROs revealing the progress of P-CP into P-Rb cells.
3. Heatmap showing the dynamic of selected Rb- and cell cycle-related genes expression in P-Rb development.


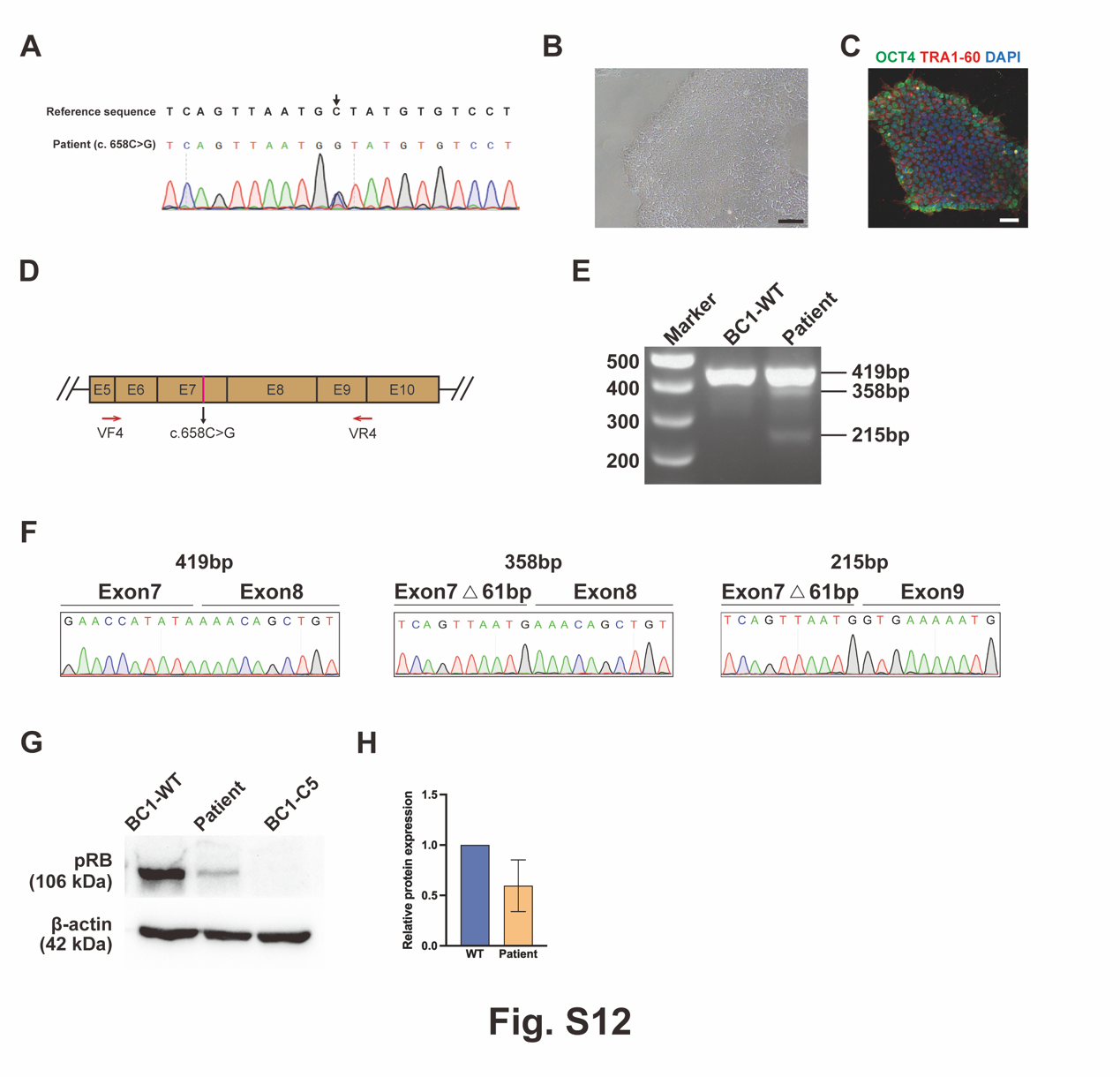


Fig. S12 Characterization of patient-specific hiPSC line and ROs with *RB1* heterozygous mutation

1. Representative bright field images of patient-specific hiPSC line.
2. Representative immunostaining images for OCT4 and TRA1-60 in patient-specific hiPSC line.
3. Sanger Sequencing of patient-specific hiPSC lines.
4. Schematic diagram of PCR primers design for analyzing c.658C>G mutation effects of the on mRNA splicing patterns.
5. RT-PCR amplification of the *RB1* transcripts obtained from patient-specific hiPSCs. Two types of additional bands were identified.
6. Sanger sequencing corresponded to WT mRNA and Two types of alternatively spliced mRNA variants from patient-specific hiPSCs.
7. Western blot analysis of pRB in WT, *RB1*^-/-^ and patient-specific hiPSC line.
8. Relative protein expression of pRB in WT hiPSC line and patient-specific hiPSC line. Data represents mean ± SD, n = 3.

Scale bars = 100 μm (B, C)


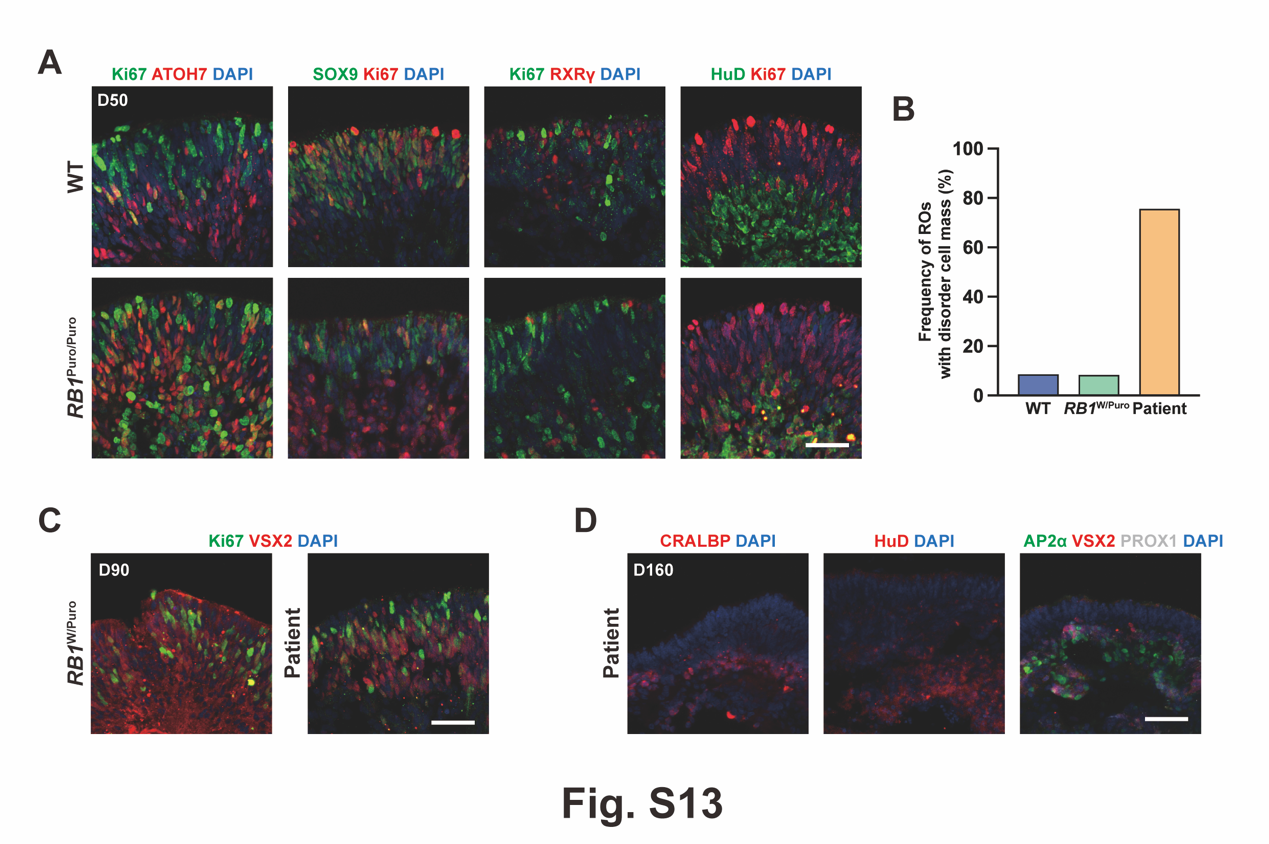


Fig. S13 Characterization of ROs with *RB1* heterozygous mutation

1. Representative immunostaining for ATOH7, SOX9, RXRγ, HuD and Ki67 in WT and *RB1*^Puro/Puro^ ROs (Gibco) at day 50.
2. Quantification for frequency of ROs with retinal lesion in different types of ROs at day 90, n = 22 (WT), n = 24 (*RB1*^W/Puro^), n = 22 (Patient).
3. Representative immunostaining for VSX2 and Ki67 in *RB1*^W/Puro^ and patient-specific ROs at day 90.
4. Representative immunostaining for CRALBP, HuD, AP2a, VSX2 and PROX1 in patient-specific ROs at day 160.

Scale bars = 50 μm (A, C, D)


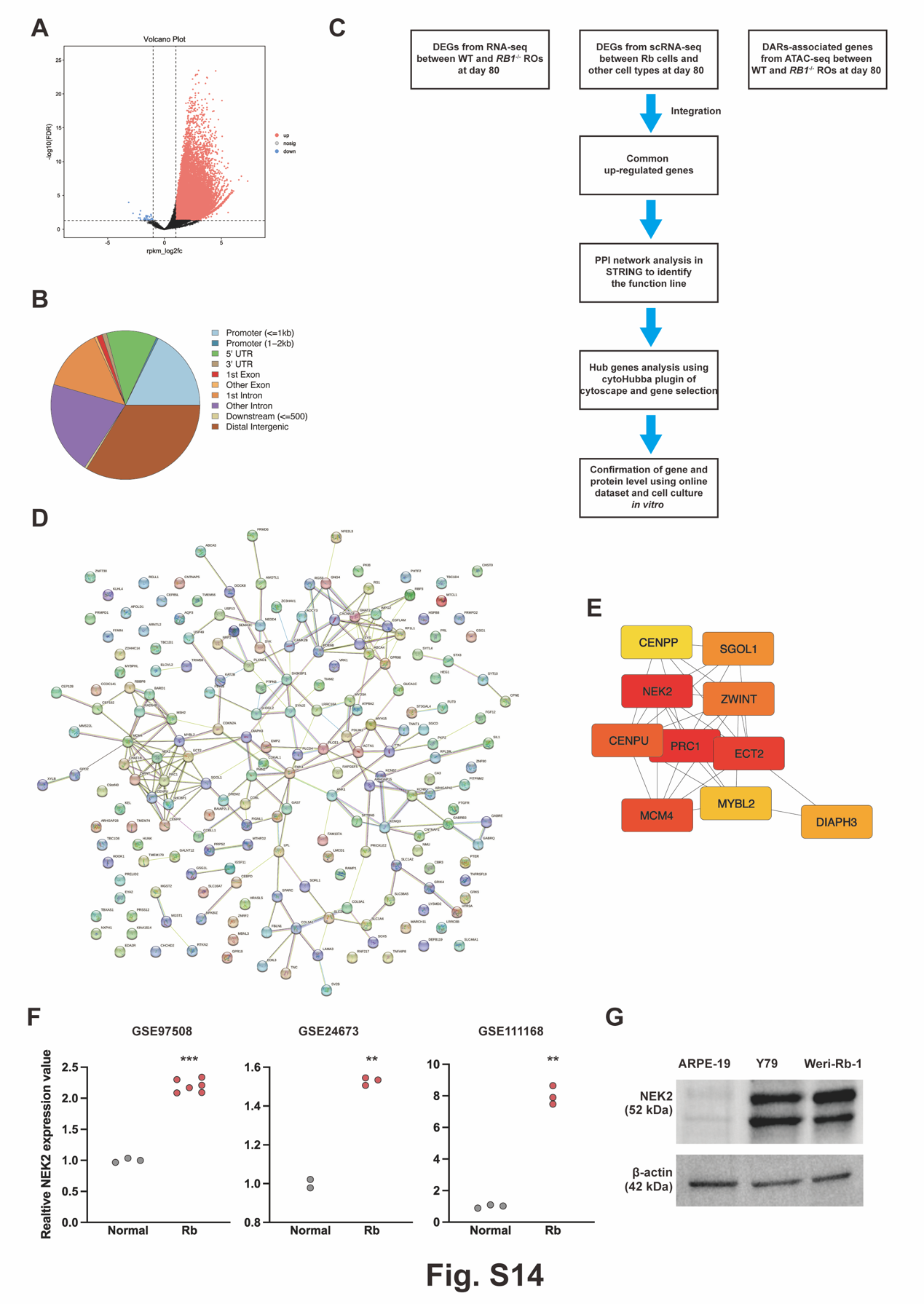


Fig. S14 Identification of NEK2 as a potential therapeutic target for Rb

1. Volcano plot visualizations of differential peaks from ATAC-seq between WT and *RB1*^-/-^ROs at day 80.
2. Genome-wide distribution of accessible chromatin regions in WT ROs.
3. Schematic illustration of integration analysis of multi-omics data to identify potential therapeutic targets.
4. Protein-protein interaction (PPI) network of collective up-regulated genes from three data sets.
5. Top ten hub genes from analysis of PPI network by the cytoHubba plugin
6. Meta-analysis of public transcriptomic data reveals consistent NEK2 overexpression in retinoblastoma versus normal retina (GSE97508/24673/111168; ****P* < 0.001, ***P* < 0.01; n = 2-6).
7. Western blot analysis of *NEK2* in Rb cell lines, Y79 and Weri-Rb-1 as well as normal retinal cell line ARPE-19.


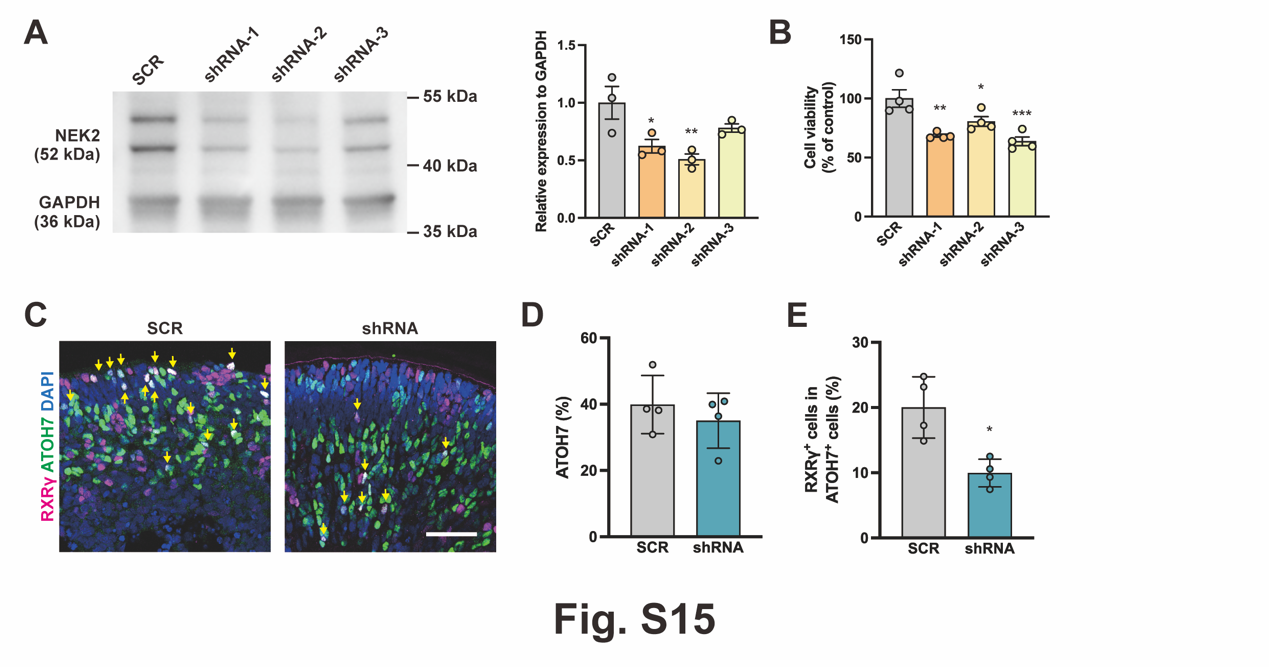


Fig. S15 Validation of the effect of NEK2 inhibition in Rb tumorigenesis by knockdown in *RB1*^-/-^ ROs

1. Western blot analysis of NEK2 after transfection of NEK2-shRNA lentivirus in Y79 cells. Data represents mean ± SD. * *P* < 0.05 vs. SCR; ** *P* < 0.01 vs. SCR, n = 3.
2. Cell viability of Y79 cells after 24 h knockdown of lentivirus with different shRNA by CCK8 assay. Data represents mean ± SD. * *P* < 0.05, ** *P* < 0.01, *** *P* < 0.001 vs. SCR, n = 4.
3. Representative immunostaining for ATOH7 and RXRγ in *RB1*^-/-^ ROs after transfection of NEK2-shRNA lentivirus and SCR-shRNA lentivirus.
4. Quantification of ATOH7^+^ cells in *RB1*^-/-^ ROs after transfection of lentivirus. Data represents mean ± SD. n = 4.
5. Quantification of RXRγ^+^ cells within ATOH7^+^ in *RB1*^-/-^ ROs after transfection of lentivirus. Data represents mean ± SD. * *P* < 0.05 vs. SCR, n = 4.

Scale bars = 50 μm (C)


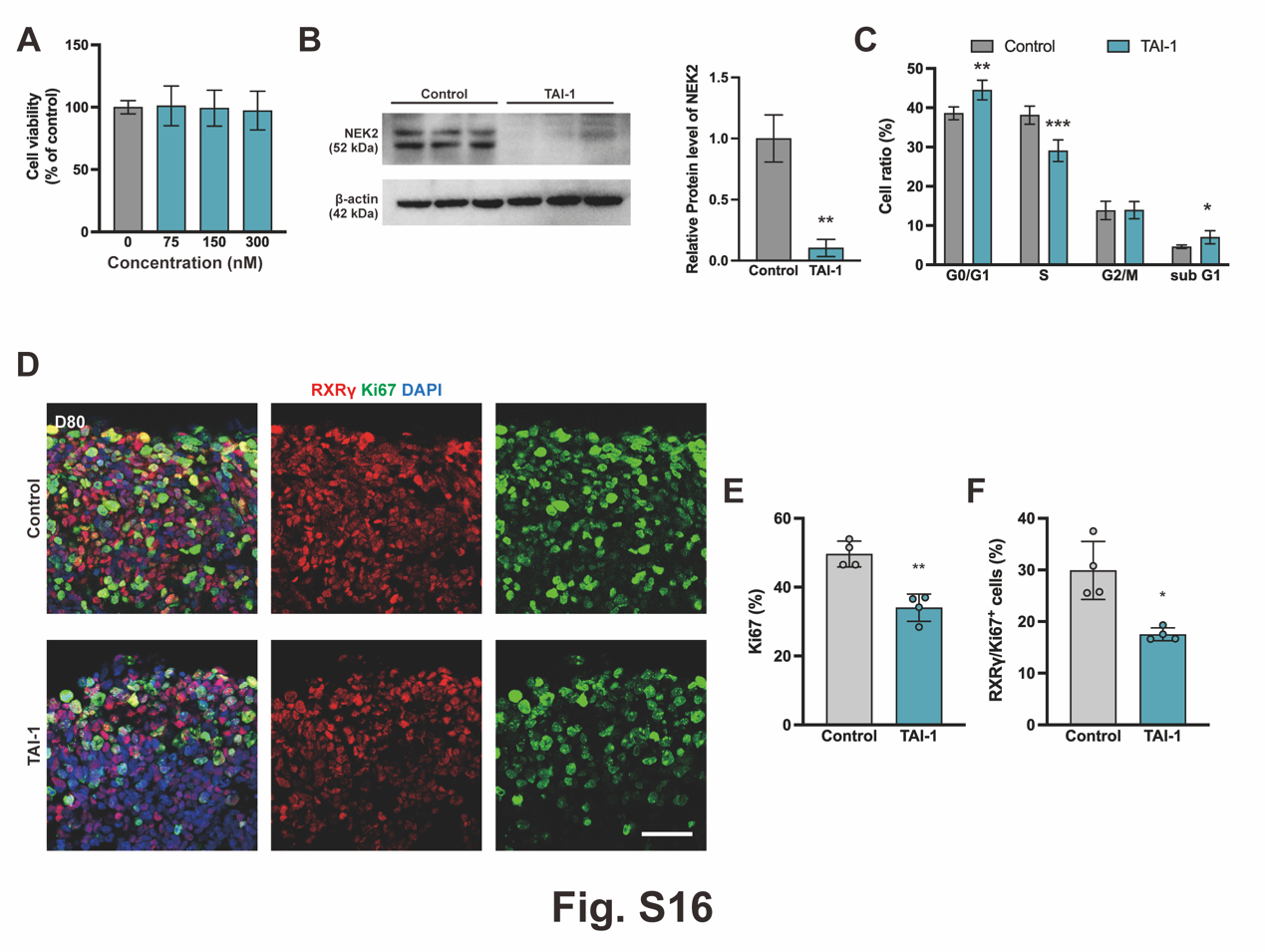


Fig. S16 Confirmation of the therapeutic effect of TAI-1 using Rb cell line and *RB1*^-/-^ ROs model

1. Cell viability of ARPE-19 after 72 h treatment with TAI-1 in different concentration by CCK8. Data represents mean ± SD, n = 6.
2. Western blot analysis of NEK2 after TAI-1 treatment in Y79 cells. Data represents mean ± SD. ** *P* < 0.01 vs. control, n = 3.
3. Cell cycle distribution of Y79 cells with TAI-1 treatment. Data represents mean ± SD. * *P* < 0.05, ** *P* < 0.01, *** *P* < 0.001 vs. control, n = 6.
4. Representative immunostaining for RXRγ and Ki67 in *RB1*^-/-^ ROs at day 80 after treatment of TAI-1.
5. Quantification of Ki67^+^ cells in *RB1*^-/-^ ROs at day 80 after treatment of TAI-1. Data represents mean ± SD. ** *P* < 0.01 vs. control, n = 4.
6. Quantification of RXRγ/Ki67^+^ cells in *RB1*^-/-^ ROs at day 80 after treatment of TAI-1. Data represents mean ± SD. * *P* < 0.05 vs. control, n = 4.

Scale bars = 50 μm (D)

Table S1 Summary of 1st xenografts using cells from WT and *RB1*^-/-^ ROs and Rb cell lines

| Cell types | Age of ROs | Number of transplanted cells | Total number of mice | Number of mice with tumor | Frequency |
| --- | --- | --- | --- | --- | --- |
| WT ROs | Day 61-85 | 1×10^5^ | 5 | 0 | 0% |
| *RB1*^-/-^ ROs | Day 67 | 1×10^5^ | 10 | 7 | 70% |
| Y79 | N/A | 1×10^5^ | 2 | 2 | 100% |
| Weri-Rb-1 | N/A | 1×10^5^ | 2 | 2 | 100% |

Table S2 Summary of 2nd xenografts using cells from 1st xenograft

| Cell types | Number of transplanted cells | Total number of mice | Number of mice with tumor | Frequency |
| --- | --- | --- | --- | --- |
| *RB1*^-/-^ ROs-derived 1st tumor | 1.2×10^5^ – 1.5×10^5^ | 5 | 5 | 100% |

Table S3

| Oligonucleotides | |
| --- | --- |
| *RB1* sgRNA Targeting Sequence | GGTGGCGGCCGTTTTTCGGG |
| Primer VF1 | TTTGTAACGGGAGTCGGGAG |
| Primer VR1 | ACCTGTCAAGTTGAAGCCGA |
| Primer VF2 | CGGCCCTGGTATTGGACAAA |
| Primer VR2 | CAAGGGTAGCGGCGAAGAT |
| Primer VF3 | ACCATGCTGATAGTGATTGTTGAA |
| Primer VR3 | CCTGTCAGCCTTAGAACCATGT |
| Primer VF4 | ACACAACCCAGCAGTTCGAT |
| Primer VR4 | ACACAACCCAGCAGTTCGAT |
| Scramble shRNA | CCTAAGGTTAAGTCGCCCTCG |
| shRNA#1 | GGGATCTGAAACCAGCCAATG |
| shRNA#2 | GCATTAATGCCTCCATTTACA |
| shRNA#3 | CCTGTATTGAGTGAGCTGAA |

Table S4: Information of antibody used in this research

| Antibodies | | |
| --- | --- | --- |
| Goat polyclonal anti-ARR3 | Novus Biologicals | NBP1-37003 |
| Mouse monoclonal anti-AP2α (3B5) | Developmental Studies Hybridoma Bank | AB_528084 |
| Rabbit polyclonal anti-ATOH7 | Novus Biologicals | NBP1-88639 |
| Goat polyclonal anti-BRN3 (C13) | Santa Cruz | sc-6026 |
| Rabbit polyclonal anti-Cleaved Caspase 3 | Cell Signaling Technology | #9661 |
| Rabbit polyclonal anti-CDKN2A/p16^INK4a^ | Bioss | bs-20656R |
| Mouse monoclonal anti-CRX(M02) Clone 4G11 | Abnova | H00001406-M02 |
| Mouse monoclonal anti-HuD(H-9) | Santa Cruz | sc-48421 |
| Mouse monoclonal anti-Ki67(B56) | BD Biosciences | 550609 |
| Rabbit polyclonal anti-Ki67 | Abclonal | A11390 |
| Rat monoclonal anti-Ki67 | Origene | TA801577S |
| Rabbit anti-L/M opsin | Gift from Dr Jeremy Nathans | N/A |
| Rabbit polyclonal anti-NANOG | Abcam | ab21624 |
| Mouse monoclonal anti-TRA-1-60 | Abcam | ab16288 |
| Mouse monoclonal anti-SSEA4 (MC813-70) | Abcam | ab16287 |
| Rabbit polyclonal anti-OCT4 | Abcam | ab19857 |
| Rabbit polyclonal anti-NEK2 | Proteintech | 14233-1-AP |
| Mouse monoclonal anti-NR2E3 | R&D Systems | PP-H7223-00 |
| Mouse monoclonal anti-NRL (F-2) | Santa Cruz | sc-374277 |
| Goat polyclonal anti-NRL | R&D Systems | AF2945 |
| Rabbit polyclonal anti-OTX1+OTX2 | Abcam | ab21990 |
| Mouse polyclonal anti-PAX6 | DSHB | AB_528427 |
| Rabbit polyclonal anti-PROX1 | Millipore | AB5475 |
| Rabbit monoclonal anti-Rb | Abcam | ab181616 |
| Mouse monoclonal anti-Rb (G3-245) | BD Biosciences | 554136 |
| Mouse monoclonal anti-Rb (4H1) | Cell Signaling Technology | #0309 |
| Rabbit polyclonal anti-RCVRN | Millipore | AB5585 |
| Mouse monoclonal anti-Rhodopsin (1D4) | Abcam | ab5417 |
| Rabbit polyclonal anti-RXRγ | Abcam | ab15518 |
| Mouse monoclonal anti-RXRγ (A-2) | Santa Cruz | sc-365252 |
| Rabbit monoclonal anti-SOX9 (ARC0190) | Abclonal | A19710 |
| Mouse monoclonal anti-STEM121 | Cellartis(Takara) | Y40410 |
| Rabbit monoclonal anti-SYK (D3Z1E) | Cell Signaling Technology | #13198 |
| Mouse monoclonal anti-SYK (4D10) | Santa Cruz | sc-1240 |
| Rabbit polyclonal anti-α-Tubulin | Beyotime | AF0001 |
| Sheep polyclonal anti-VSX2 | Millipore | AB9016 |
| Donkey Anti-Mouse IgG-488 | Invitrogen | A21202 |
| Donkey Anti-Rabbit IgG-488 | Invitrogen | A21206 |
| Donkey Anti-Rat IgG-488 | Invitrogen | A21208 |
| Donkey Anti-Mouse IgG-555 | Invitrogen | A31570 |
| Donkey Anti-Rabbit IgG-555 | Invitrogen | A31572 |
| Donkey Anti-Goat IgG-555 | Invitrogen | A21432 |
| Donkey Anti-Sheep IgG-555 | Invitrogen | A21436 |
| Donkey Anti-Rabbit IgG-647 | Invitrogen | A31573 |
| Donkey Anti-Mouse IgG-647 | Invitrogen | A31571 |
