# Supplementary figures and images for: Longitudinal analysis of retinal cell state transitions in RB1-deficient retinal organoids reveals the nascent cone precursors are the earliest cell-origin of human retinoblastoma
Source: Cell Death Dis. 2026 Jan 14;17(1):34. doi: 10.1038/s41419-025-08191-x (PMC12805002; doi:10.1038/s41419-025-08191-x)

Fig. 1F


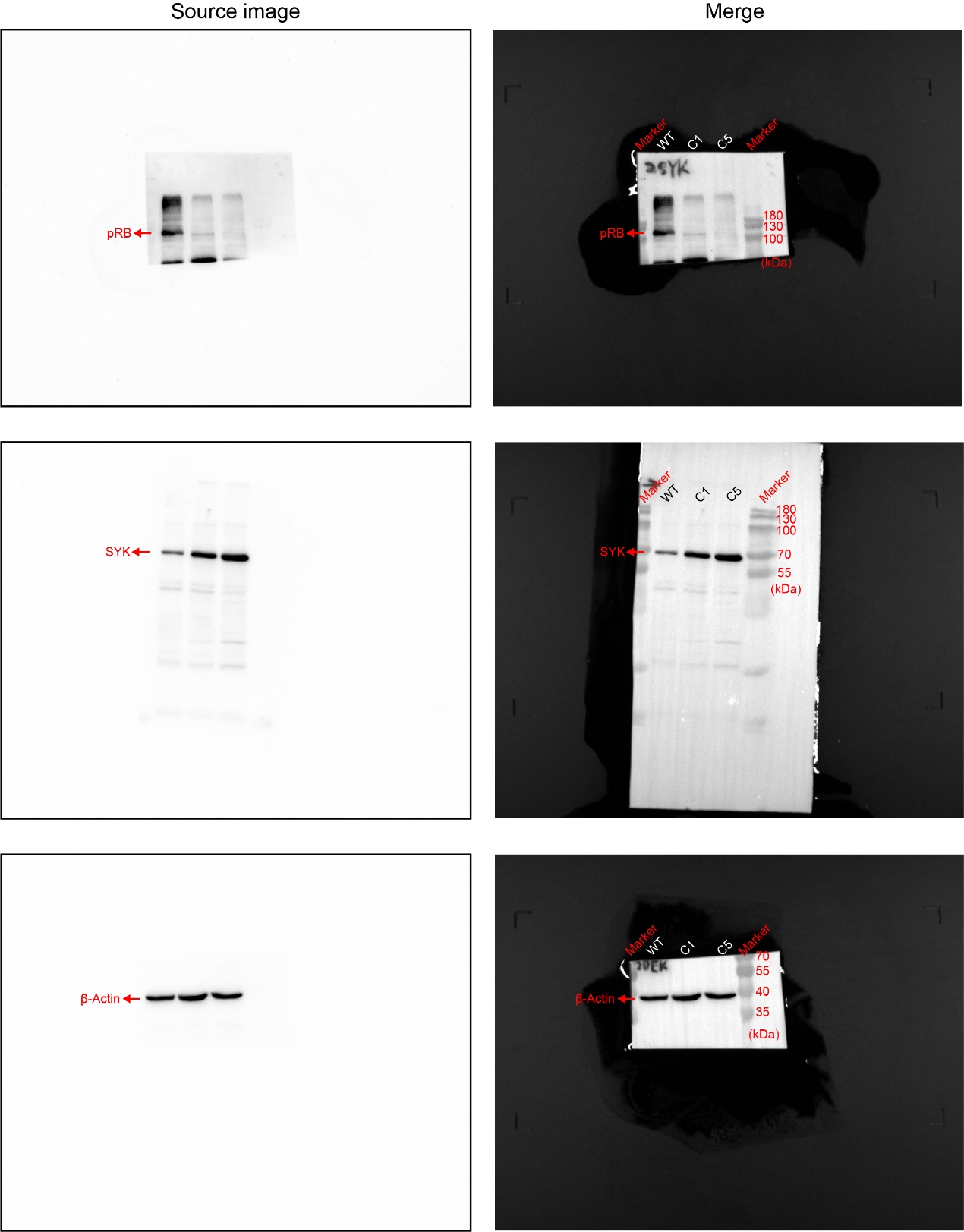


Fig. S1D


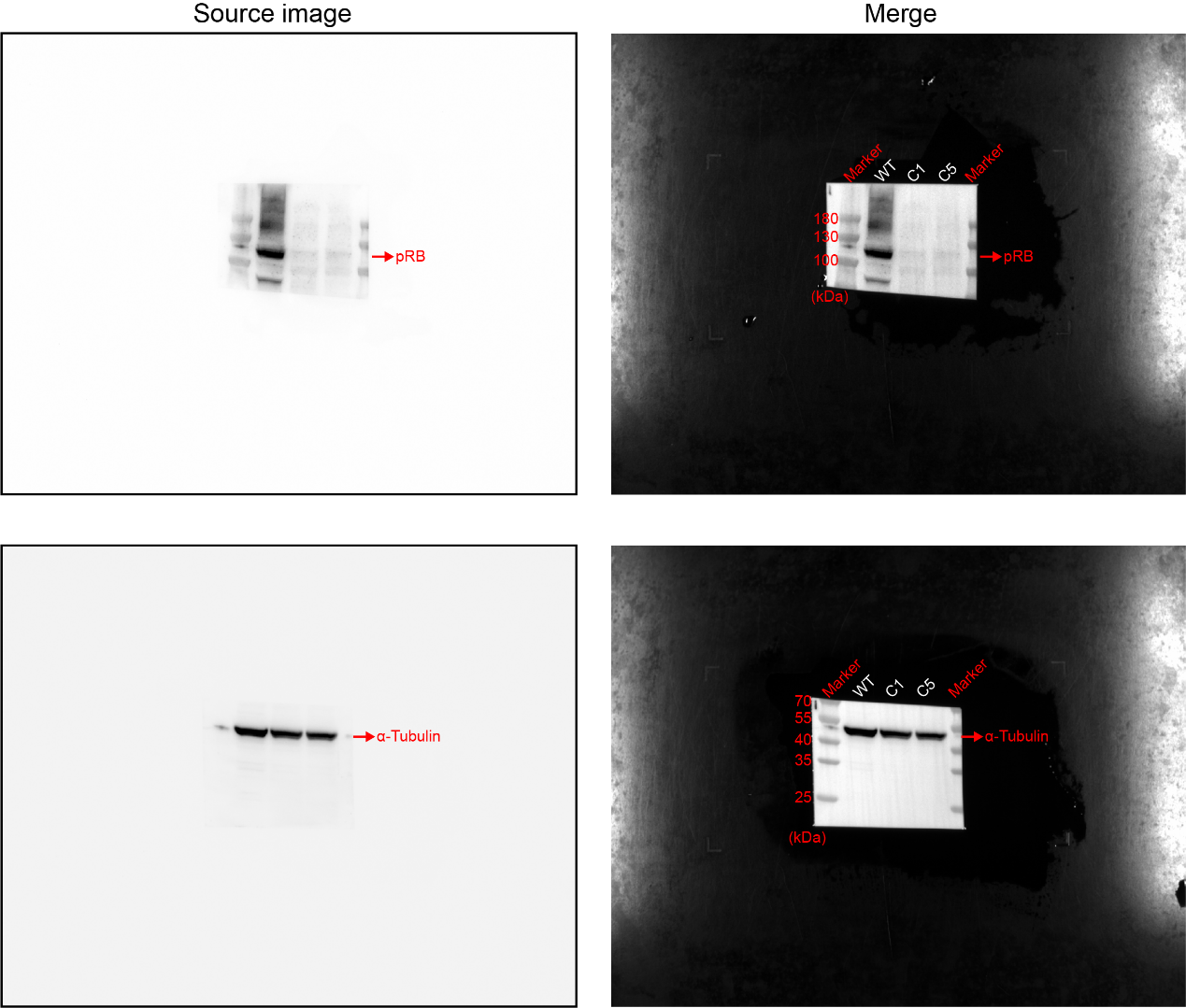


Fig. S3D


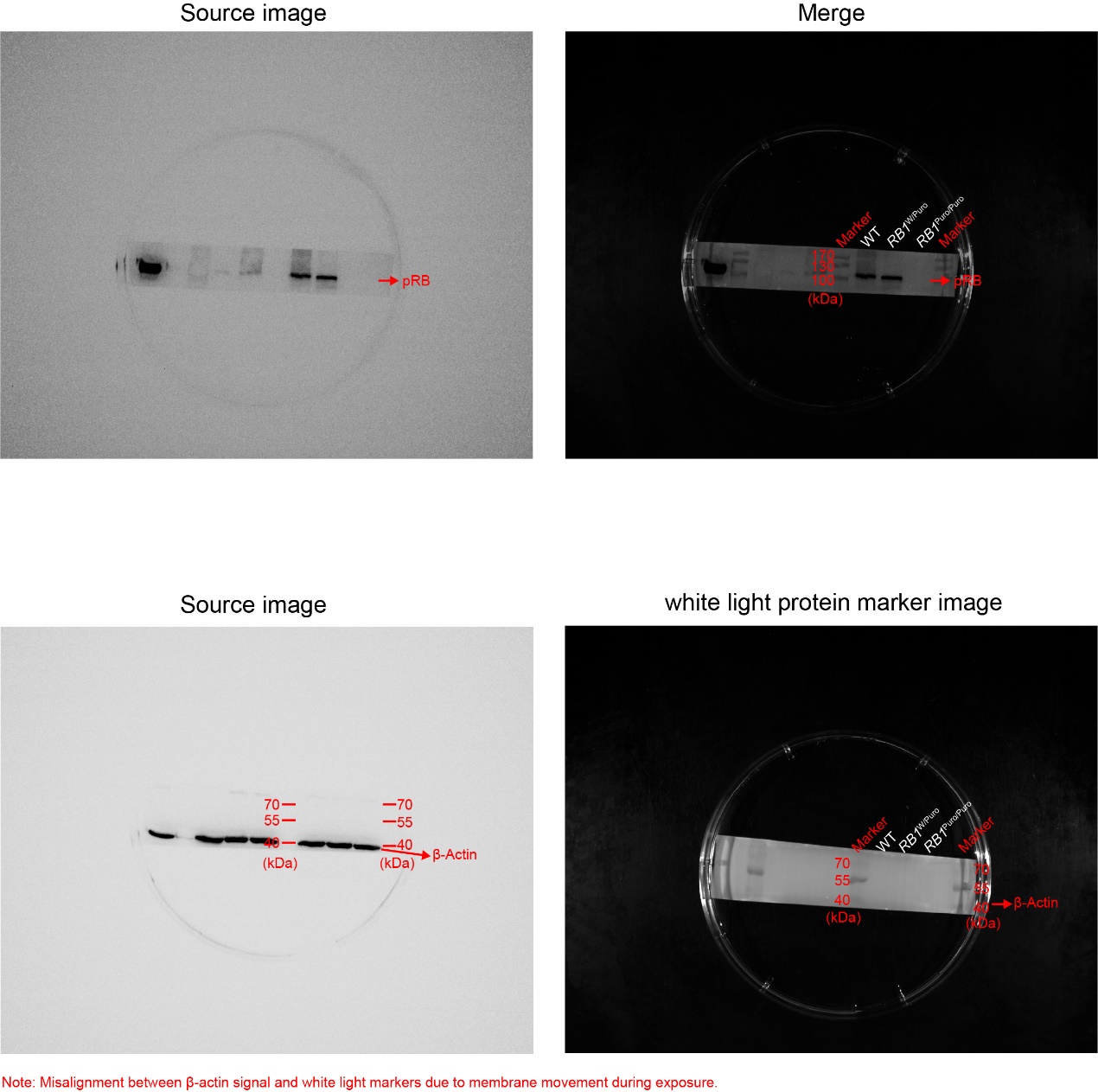


Fig. S12G


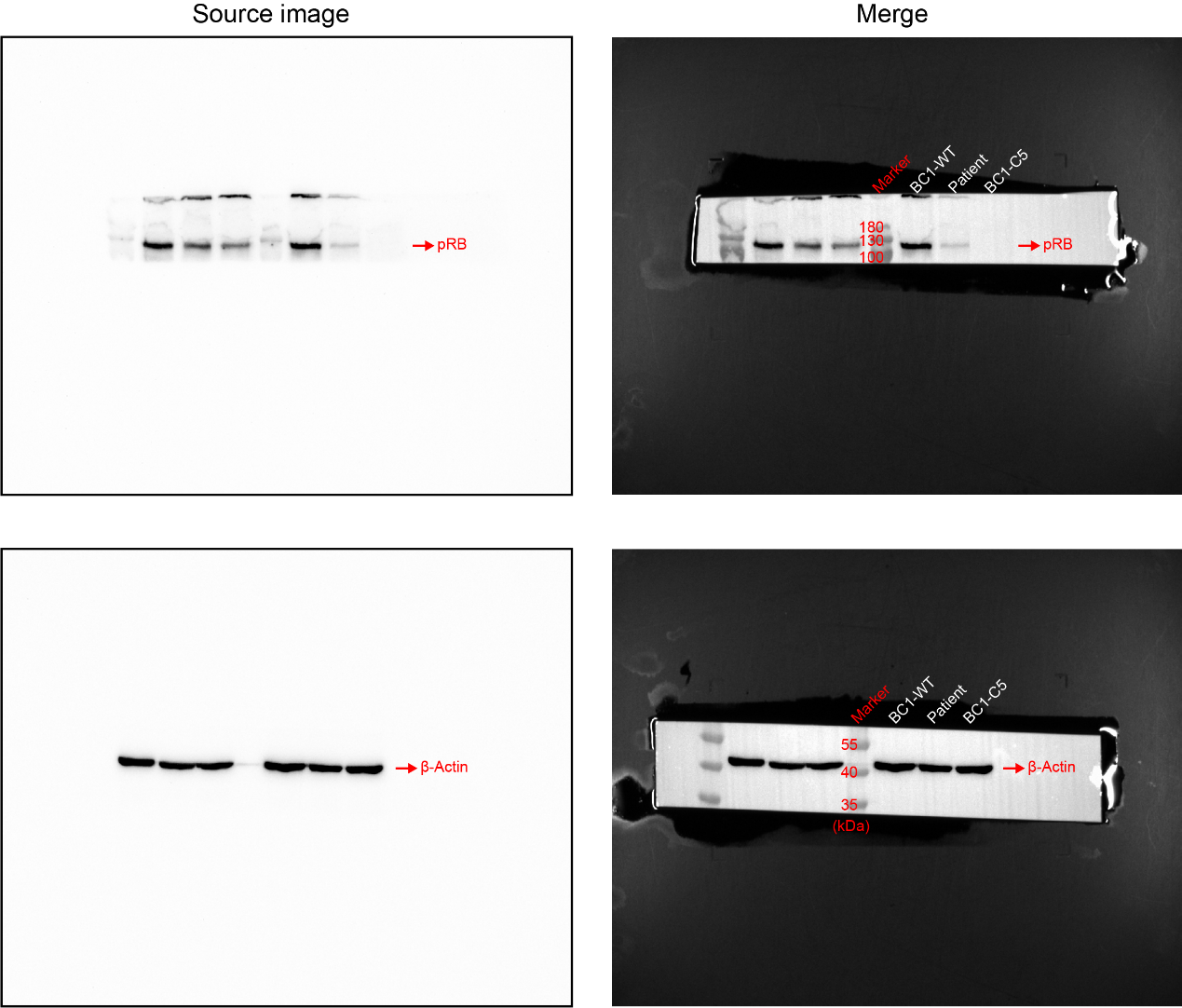


Fig. S14G


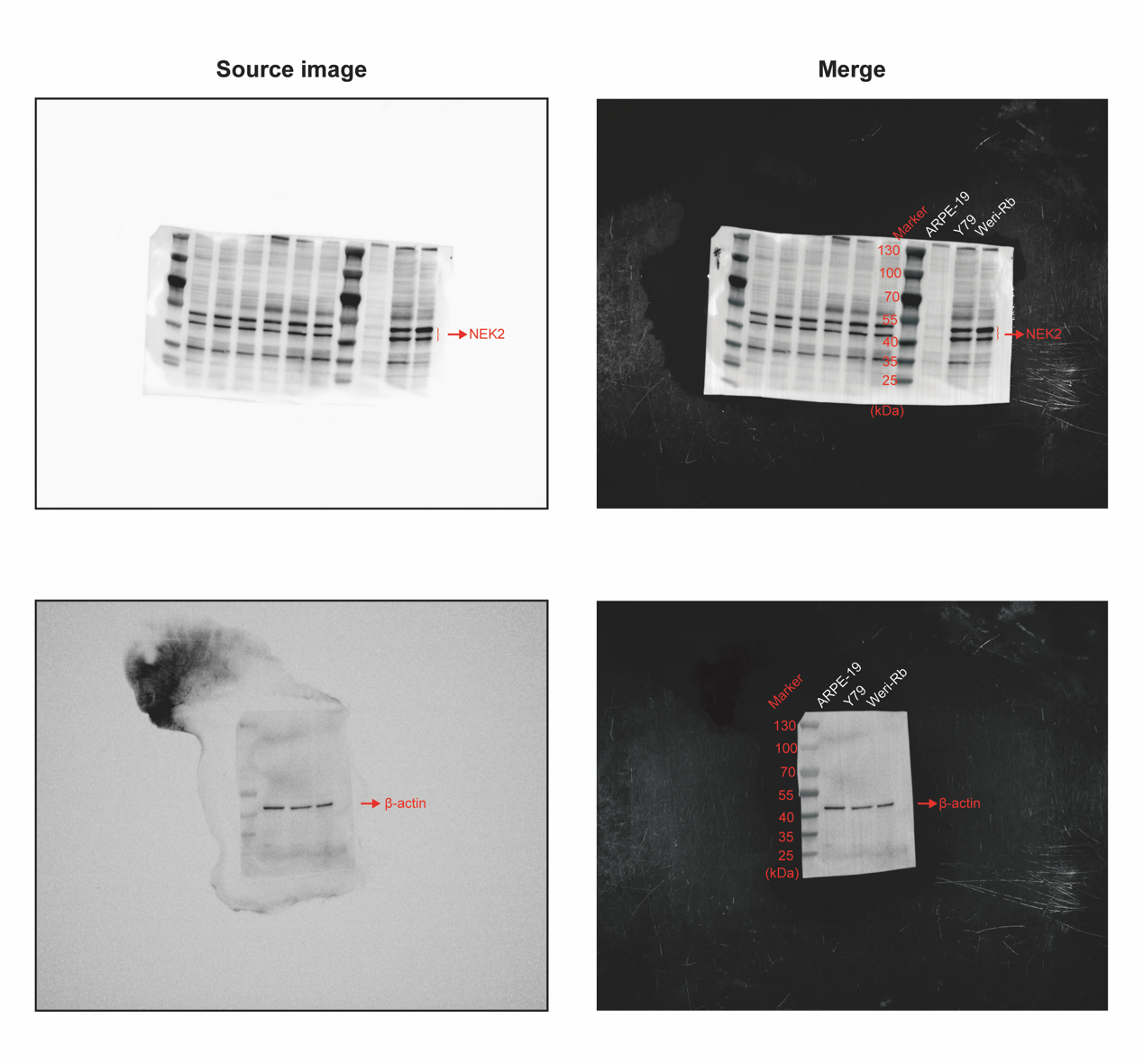


Fig. S15A


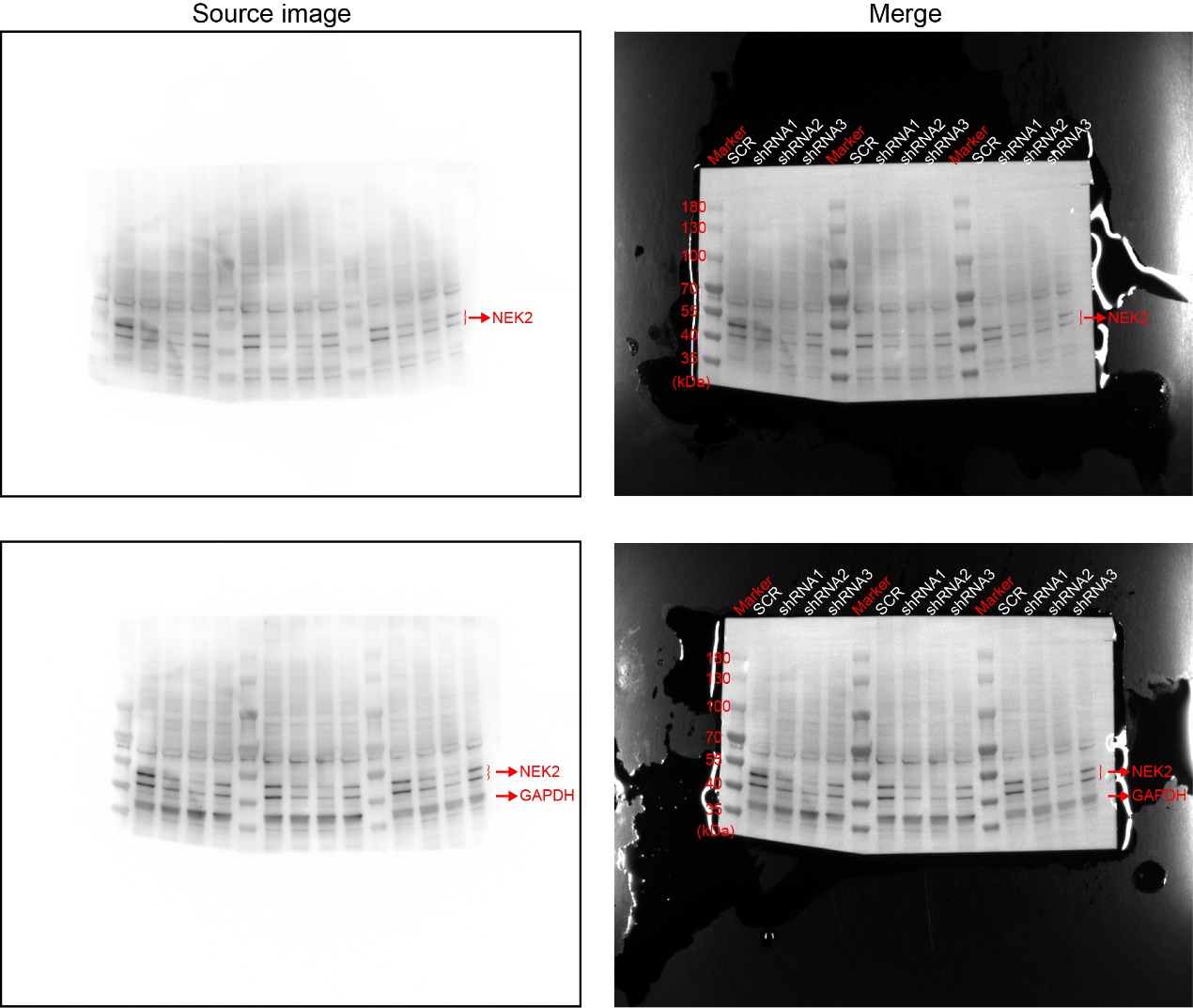


Fig. S16B


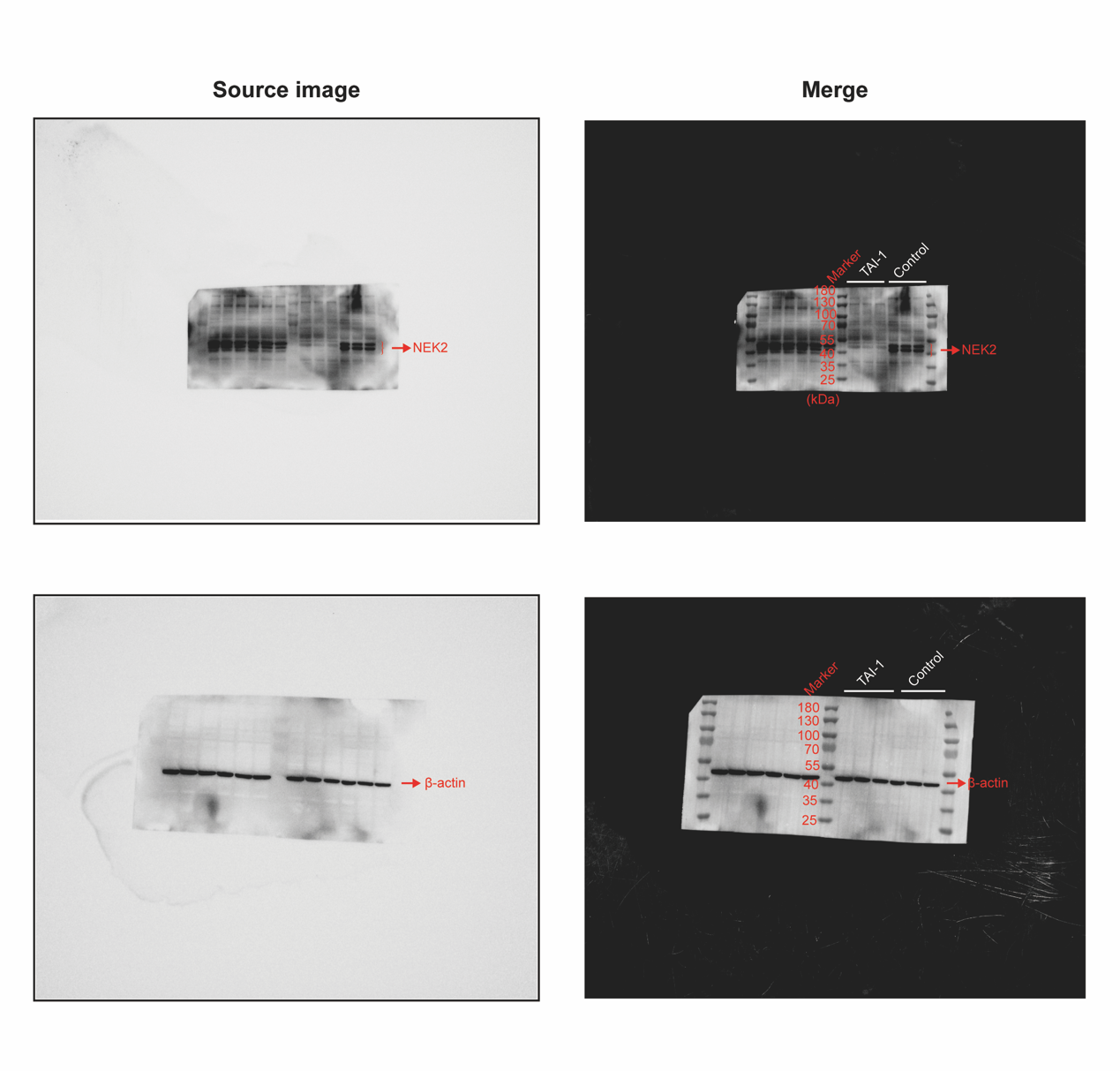

Supplement: Supplementary file 2 — Original data [file 41419_2025_8191_MOESM2_ESM.docx]
